# Paraventricular nucleus–locus coeruleus VGlut2 neural circuit regulates energy metabolism in mice

**DOI:** 10.1038/s41419-025-08238-z

**Published:** 2025-12-09

**Authors:** Haodong Liu, Penghui Li, Mingyang Yu, Xin Zhang, Hefeng Zhu, Yang He, Zelin Zhang, Xiaolong Li, Bingkun Teng, Jiaxin Fan, Wenchao Yang, Junzhe Yin, Qinqin Hao, Guifang Cao, Haijun Li, Shuying Liu, Yongqiang Li, Junguang Ren, Yujie Chen, Chenguang Du

**Affiliations:** 1 College of Veterinary Medicine, Inner Mongolia Agricultural University, Hohhot, Inner Mongolia China; 2 Inner Mongolia Autonomous Region Key Laboratory of Veterinary Basic and Epidemic Prevention and Control of Herbivorous Livestock, Hohhot, China; 3 Inner Mongolia Huimu Animal Husbandry Co., Ltd., Inner Mongolia, Xing’an league, China; 4 Inner Mongolia Grassland Black–bone Sheep Biological Technology Co., Ltd., Inner Mongolia, Hohhot, China; 5 Vocational and Technical College, Inner Mongolia Agricultural University, Baotou, Inner Mongolia China

**Keywords:** Homeostasis, Obesity

## Abstract

The role of the central nervous system in energy homoeostasis remains unclear. This study examined the role of VGlut2-expressing neurons in the paraventricular nucleus of the hypothalamus (PVH^VGlut2^) and their downstream circuits in the regulation of energy homoeostasis. Long-term high-fat diet (HFD) disrupts energy balance and compensatorily activates PVH^VGlut2^ neurons that innervate interscapular brown adipose tissue (iBAT). These neurons are inhibited during food consumption, suggesting their involvement in feeding and energy metabolism regulation. Activation of PVH^VGlut2^ neurons reduces food intake and enhances iBAT thermogenesis. Further, optogenetic PVH^VGlut2^→ locus coeruleus (LC) circuit activation inhibited feeding and elevated iBAT temperature, which was blocked by sympathetic nerve denervation. Long-term chemogenetic PVH^VGlut2^ → LC neural circuit activation ameliorates HFD-induced obesity and insulin resistance. The PVH^VGlut2^ → LC circuit integrates feeding inhibition and peripheral thermogenesis signals to regulate energy metabolism, offering potential intervention targets for obesity and other energy homoeostasis disorders.

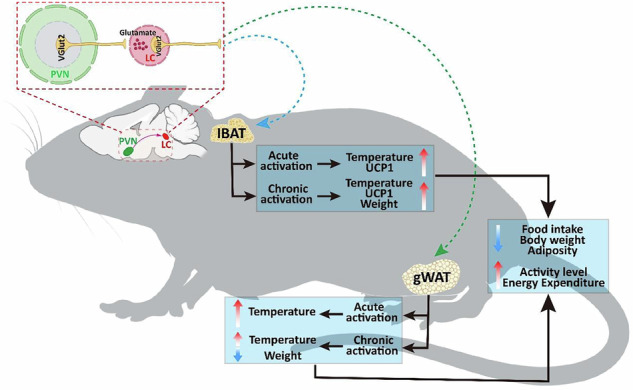

## Introduction

Obesity, a global health crisis, is primarily driven by a long-term imbalance between energy intake and expenditure [1, 2]. The hypothalamus plays a central role in this process by integrating peripheral metabolic signals to regulate energy homoeostasis. The nervous system dynamically regulates energy balance by responding to peripheral signals, such as leptin and insulin [3]. However, in diet-induced obesity (DIO), this regulatory mechanism becomes impaired. For instance, the inhibitory effect of intracerebroventricular leptin injection on high-fat diet (HFD) intake is compromised [4], suggesting that specific nuclei develop resistance to metabolic signals [5]. This resistance may stem from abnormal adenosine A1 receptor (A1R) regulation in the paraventricular nucleus of the hypothalamus (PVH) [6]. HFD upregulates A1R expression, inhibiting cAMP/PKA signalling and inducing glial inflammation and glutamate dysregulation [7]. The PVH integrates various peripheral metabolic signals [8], receiving projections from arcuate nucleus (ARC) neuropeptide Y/agouti-related protein (NPY/AgRP) and pro-opiomelanocortin (POMC) neurons, which regulate food intake by inhibiting or activating PVH neurons via gamma-aminobutyric acid (GABA) [9]. This bidirectional regulatory feature makes the PVH a key target for metabolic research.

Glutamatergic neuron marker vesicular glutamate transporter 2 (VGlut2) [10] is expressed throughout the brain, including the PVH (PVH^VGlut2^) [11]. VGlut2 regulates energy metabolism and interscapular brown adipose tissue (iBAT) heat production through a multi-brain region synergistic mechanism. VGlut2 neuron activation in the preoptic area (POA) reduces core temperature and energy expenditure, dependent on glutamatergic projections from the POA to the lateral parabrachial nucleus (PBN) [12]. Under heat stimulation, this pathway induces caudal vasodilation and inhibits iBAT heat production [13]. Notably, VGlut2 neurons in the locus coeruleus (LC) project to the PVH, integrating metabolic signals to regulate energy balance [14]. Chemogenetic activation of the LC glutamatergic pathway can inhibit food intake and reduce body weight [15]. Additionally, noradrenergic neurons in the LC project to the PBN to inhibit hunger signals and activate iBAT via spinal presympathetic neurons [16, 17]. Despite these advances, the specific role of VGlut2-expressing neurons in PVH-LC interactions remains poorly understood. Although anatomical studies have identified neural connections between the PVH and LC, the functional dynamics and physiological significance of the PVH^VGlut2^ → LC circuit in energy metabolism have not yet been systematically investigated.

In this study, we employed viral tracing, optogenetics, chemogenetics, fibre photometry, and pharmacological blockade of glutamate release to investigate the role of PVH^VGlut2^ neurons and the PVH^VGlut2^ → LC circuit in energy metabolic homoeostasis. Our findings reveal a critical neural pathway linking hypothalamic and brainstem centres in the regulation of feeding behaviour, thermogenesis, and energy balance.

## Materials and Methods

### Experimental animals

Adult male VGlut2^Cre/Cre^ (JAX# 016962), VGAT^Cre/Cre^ (JAX# 028862), and Rosa26-LSL-tdTomato (JAX# 007914) transgenic mice on a C57BL/6 background (8–12 weeks old) were used in this study. All transgenic mice were purchased from The Jackson Laboratory (Bar Harbour, ME, USA).

Mice were housed under controlled conditions (22–24 °C. 50–60% relative humidity, 12-h light/dark cycle; lights on at 07:00) with *ad libitum* access to food and water unless otherwise specified. All experimental procedures were reviewed and approved by the Animal Welfare and Experimental Animal Ethics Committee of Inner Mongolia Agricultural University (Approval number: NND2022203). All guidelines and regulations regarding animal breeding and the use of relevant institutions and the state were followed.

### Stereotaxic surgery

Mice were anesthetised with 2% isoflurane and fixed on a stereotaxic apparatus (Neurostar). Their body temperature was maintained at 37 °C using a heating pad. A 5 μL Hamilton microsyringe was used to inject the virus (0.3 μL per site, titre: 1 × 10^12^ vg/mL) into the PVH (coordinates: anteroposterior [AP] −0.58 mm, mediolateral [ML] ±0.25 mm, dorsoventral [DV] −4.9 mm) or the LC (coordinates: AP − 5.04 mm, ML ± 0.98 mm, DV − 3.8 mm) at a rate of 0.1 μL/min. Following the injection, the needle was left in place for 10 min to prevent backflow. Mice were then placed in an incubator at 37 °C for recovery and subsequently returned to their original cages.

The following adeno-associated virus (AAV) vectors were used in this study: AAV9-DIO-GCaMP6f (BrainVTA, Cat# PT-0106), AAV9-DIO-EGFP (BrainVTA, Cat# PT-0168), AAV2/9-DIO-ChR2-EGFP (Taitool, Cat# S0858-9), AAV2/9-DIO-NpHR-mCherry (BrainVTA, Cat# PT-0628), AAV2/9-DIO-mCherry (BrainVTA, Cat# PT-0013), AAV1-DIO-mCherry (Addgene, Cat# 50459), AAV2/9-DIO-hM3D-EGFP (BrainVTA, Cat# PT-0891), AAV2/9-DIO-hM4D-EGFP (BrainVTA, Cat# PT-0987), AAV1-CaMKIIα-Cre (BrainVTA, Cat# PT-0220), AAV2/9-DIO-ChR2-EYFP (BrainVTA, Cat# PT-0001), AAV2/9-DIO-EYFP (HANBIO, Cat# PT-0012), AAV1-DIO-hM3D-mCherry (BrainVTA, Cat# PT-0019), AAV1-DIO-hM4D-mCherry (BrainVTA, Cat# PT-0020), AAV/R-DIO-ChR2-EGFP (BrainCase, Cat# BC-0687), AAV2/9-fDIO-hM3D-mCherry (Creative Biolabs, Cat# ZP119), AAV2/9-fDIO-mCherry (BrainVTA, Cat# PT-0339), AAV/R-DIO-Flpo (Creative Biolabs, Cat# ZP118), AAV2/9-fDIO-TeNT-EGFP (BrainVTA, Cat# PT-2434), AAV2/9-fDIO-EGFP (BrainVTA, Cat# PT-0435), AAV/R-DIO-hM3D-mCherry-Flpo (BrainVTA, Cat# 57070645).

## Results

### HFD causes weight gain and metabolic disorders in mice

To systematically analyse the metabolic phenotype differences between two dietary patterns, HFD and standard chow, male C57BL/6 J mice were used. Within 6 weeks, mice in the HFD group exhibited a significant increase in body weight (*p* = 0.0097) (Fig. S1A). Furthermore, glucose tolerance (*p* = 0.0246, *p* = 0.0007) and insulin sensitivity (*p* = 0.0264, *p* < 0.0001) (Fig. S1B and C) were lower in HFD-fed mice than those in the control group, indicating impaired glucose metabolism. Concurrently, their iBAT weight decreased, whereas both gonadal white adipose tissue (gWAT) and liver weights increased significantly (*p* = 0.0349, *p* = 0.0036, *p* = 0.0001, respectively) (Fig. S1D). Thermometry results also showed decreased iBAT (*p* = 0.0444) and overall body (*p* = 0.0018) temperatures (Fig. S1E) in the HFD group. Collectively, these findings demonstrate that HFD profoundly disrupts energy homoeostasis.

### HFD alters c-Fos neuron activity in murine PVH

To identify hypothalamic nuclei related to HFD-induced obesity, c-Fos immunohistochemistry was used to compare neuronal activity in hypothalamic nuclei between diet-induced obese (DIO) and metabolically normal (lean) mice under fasting and free-feeding conditions.

**Fig. 1:**
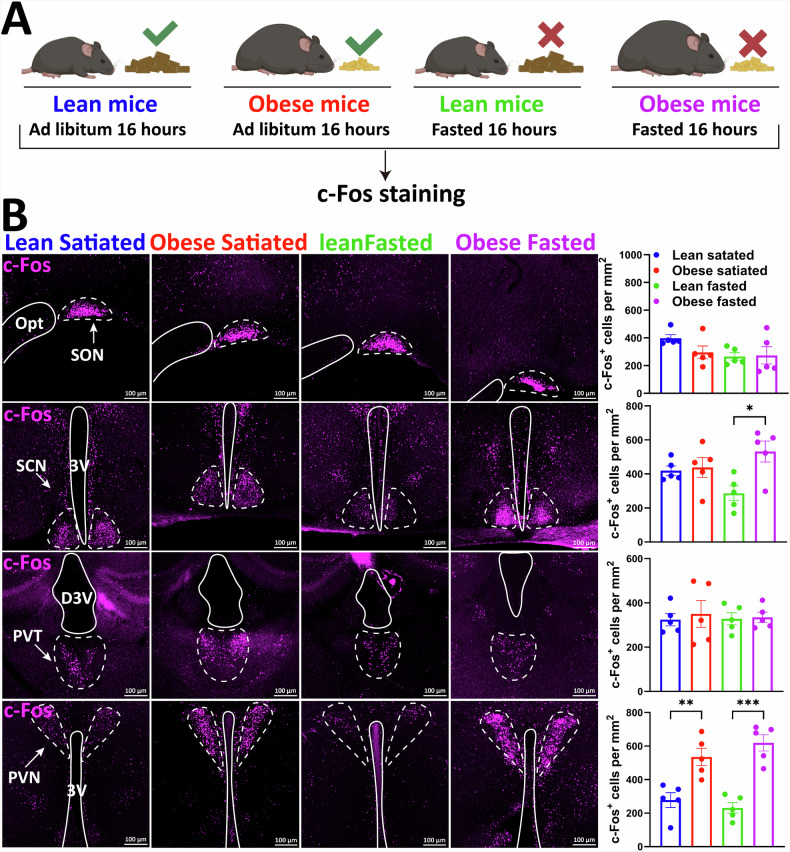
c-Fos expression analysis in lean and obese mice under different dietary conditions. **A** Schematic diagram of the experimental design. **B** c-Fos staining results under different dietary conditions, with representative images of different brain regions (Opt, SON, PVT, and PVH) shown on the left and c-Fos-positive cell counts in each brain region on the right. *n* = 5 mice per group. Two-way ANOVA and Šídák’s test (left). Data are presented as mean ± standard error of mean (s.e.m). PVH, paraventricular nucleus of the hypothalamus.

No significant change was observed in the number of c-Fos neurons within the supraoptic nucleus or paraventricular thalamic nucleus between DIO and normal mice in fasting and satiated states. However, in the fasting state, the number of c-Fos neurons in the suprachiasmatic nucleus of DIO mice increased when compared with that of normal mice (*p* = 0.0165) (Fig. 1A). Notably, the number of c-Fos neurons in the PVH of obese mice was consistently higher than that of normally fed mice, regardless of whether in the fasting or refeeding state (*p* = 0.0046, *p* = 0.0100) (Fig. 1B). Therefore, HFD-induced obesity may affect body weight regulation by altering the activation state of specific hypothalamic regions. Among these, abnormal changes in PVH neuron activity may play a crucial role in the increased body weight observed in obese mice.

### VGlut2 neuron identification in the PVH→iBAT pathway in the HFD model

**Fig. 2:**
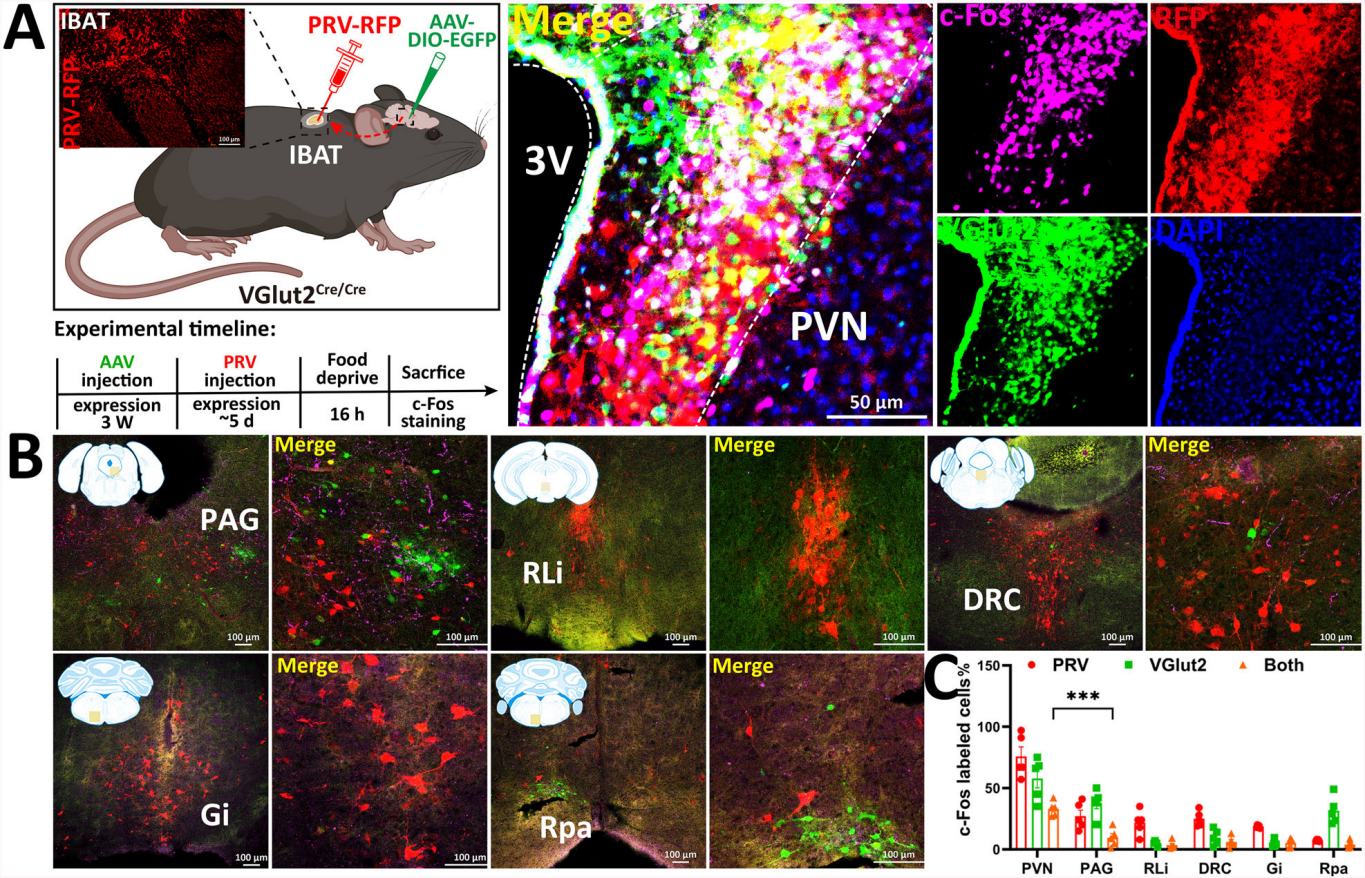
Viral tracing reveals the multisynaptic circuit of VGlut2 neurons in the fasting-activated state. **A** Schematic diagram of PRV injection into iBAT of diet-induced obese mice (left). PVH virus expression (right). **B** Distribution of fluorescent signals in the midbrain and brainstem. **C** Quantitative analysis of the total number of upstream neurons labelled by PRV, c-Fos, and VGlut2. *n* = 5 mice. Two-way ANOVA, Šídák’s test. Data are presented as mean ± standard error of mean (s.e.m). iBAT dorsoscapular brown adipose tissue, PVH paraventricular nucleus of the hypothalamus.

A characteristic feature of HFD-induced obese mice is their lower iBAT temperature compared to that of the control group (Fig. S1E), indicating impaired thermogenic function and reduced energy consumption. To analyse the specific neural projection pathway from PVH^VGlut2^ → iBAT and to identify the types of PVH neurons affected by HFD intervention, a VGlut2^Cre/Cre^ mouse model was used. This was combined with retrograde trans-multi-synaptic virus tracing technology for circuit analysis and PVH neuron type identification (Fig. 2A).

Strong pseudorabies virus (PRV) labelling was observed in various brain regions, including the periaqueductal gray, rostral linear nucleus, dorsal raphe nucleus, gigantocellular reticular nucleus, and raphe pallidus nucleus (Fig. 2B). Notably, VGlut2/PRV/c-Fos triple-labelled signals were detected within the PVH. The proportion of PRV and VGlut2 neurons in the PVH was highest. Moreover, the proportion of c-Fos-labelled cells co-localised with VGlut2/PRV in the PVH was significantly higher than that in other brain regions (*p* = 0.0005) (Fig. 2C).

These findings suggest that the PVH contains VGlut2 neurons closely related to DIO and iBAT thermogenesis.

### PVH^VGlut2^ neuron activity is associated with feeding behaviour

**Fig. 3:**
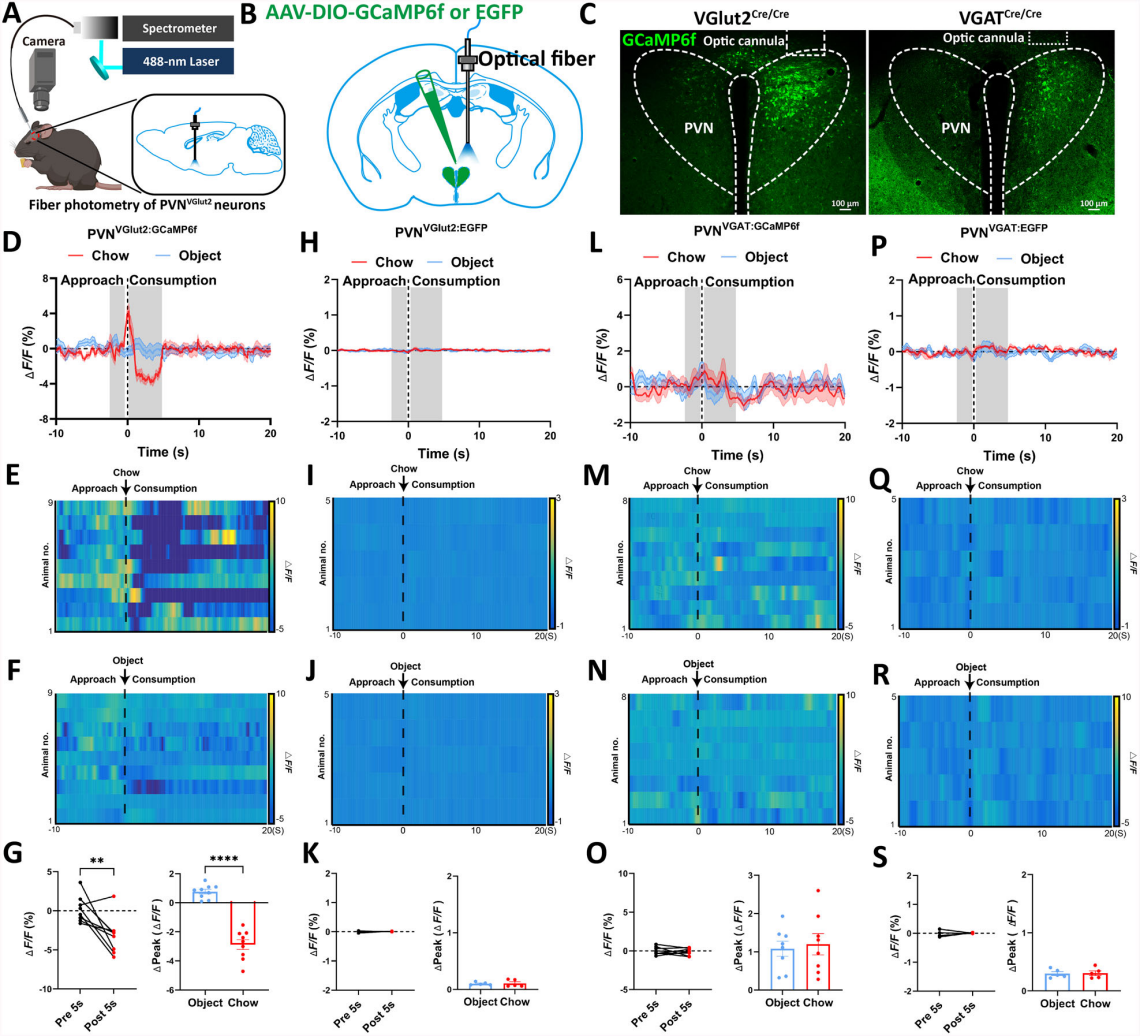
Changes in PVH^VGlut2^ and PVH^VGAT^ neuron activity during feeding. **A** Schematic diagram illustrating the recording PVH GCaMP neuron activity in mice. **B** Schematic diagram of AAV-DIO-GCaMP6f-EGFP and control virus injection into the PVH. **C** Representative images showing GCaMP6f expression and fibre optic implantation in VGlut2^Cre/Cre^ mice (left) and VGAT^Cre/Cre^ mice (right). **D**–**G** Average stimulus frequency histograms of Ca^2+^ signals in VGlut2 neurons. **D** Heatmaps of individual animal Ca^2+^ (ΔF/F) responses before and after the onset of food ingestion (**E**) and object sniffing (**F**) Average Ca2+ (ΔF/F) responses during food ingestion and object sniffing. Dot plot representing the average ΔF/F (%) for each mouse (**G**, left), and a bar graph showing the average Δpeak ΔF/F (**G**, right). **H**–**K** Average peri-stimulus histograms of EGFP signals in VGlut2 neurons. (**H**) Heatmaps of individual animal EGFP (ΔF/F) signals (**I**) and average EGFP ΔF/F signals during food consumption (**J**) and object sniffing. The average ΔF/F (%) for each mouse is presented as a dot plot (**K**, left), and the average Δpeak ΔF/F is presented as a bar graph (**K**, right). **L**–**O** Similar to **D**–**G**, but recorded in VGAT^Cre/Cre^ mice. **P**–**S**, Similar to **H**–**K**, but recorded in VGAT^Cre/Cre^ mice. VGlut2:GcaMP6f *n* = 9 mice, VGlut2:EGFP *n* = 5 mice, VGAT:GcaMP6f *n* = 8 mice, VGAT:EGFP *n* = 5 mice, Unpaired t-test. Data are presented as mean ± standard error of mean (s.e.m). PVH, paraventricular nucleus of the hypothalamus.

To reveal PVH^VGlut2^ neuronal activity patterns, fibre photometry was used to monitor VGlut2 neurons in VGlut2^Cre/Cre^ mice during foraging and feeding (Fig. 3A and B). AAV-DIO-GCaMP6f showed specific expression in the PVH of both *VGlut2*^Cre/Cre^ and *VGAT*^Cre/Cre^ mice, confirming the successful viral targeting (Fig. 3C). Ca^2+^ signals of PVH^VGlut2^ neurons significantly increased during the foraging stage and were rapidly inhibited during feeding (Fig. 3D–F). In contrast, Ca^2+^ signals remained unchanged when mice interacted with non-food objects (Fig. 3H–J). Thus, VGlut2 neuron activity is closely related to feeding behaviour.

To further analyse the regulatory relationship between feeding-related behaviours and PVH^VGlut2^ neuron activity, the Ca^2+^ signals for each mouse were averaged. The Ca^2+^ signals of VGlut2 neurons immediately increased at the start of foraging. Crucially, during food consumption, the endogenous activity of PVH^VGlut2^ neurons was significantly inhibited (*p* = 0.0016; *p* < 0.0001) (Fig. 3G–K). In VGAT^Cre/Cre^ mice, no notable changes in Ca^2+^ signals were captured during feeding or when interacting with objects (*p* = 0.4481, *p* = 0.4621) (Fig. 3L–S), suggesting that PVH^VGAT^ neurons may not be involved in regulating feeding behaviour.

### Feeding and thermogenesis regulation by PVH^VGlut2^ neurons

**Fig. 4:**
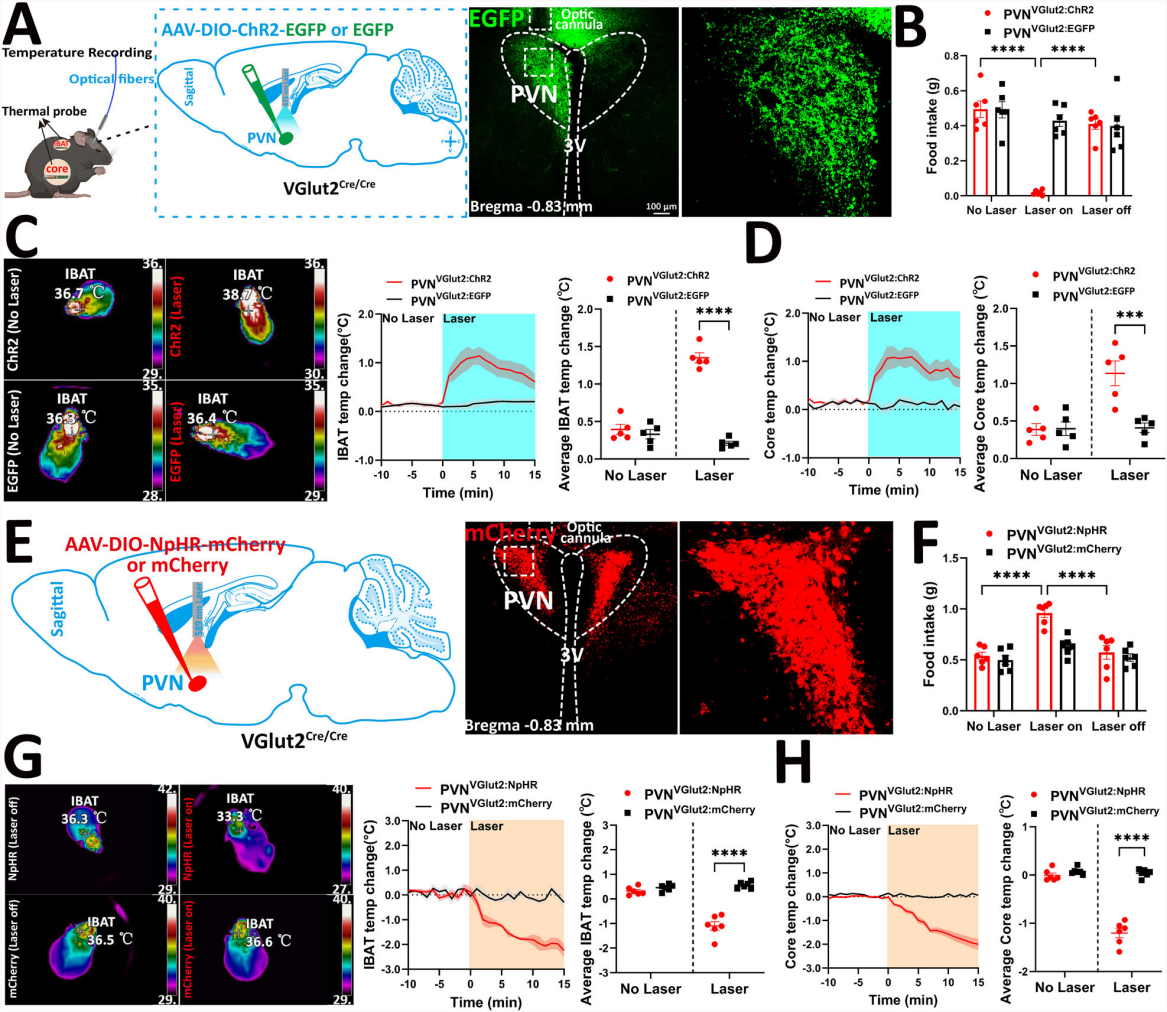
Optogenetic PVH^VGlut2^ stimulation bidirectionally regulates feeding behaviour and iBAT heat production. **A** Schematic diagram representing optogenetic activation (left), ChR2 expression in PVH^VGlut2^ neurons (right). **B** Food intake of fasting mice before and after optogenetic activation of PVH^VGlut2^ neurons. **C** Representative thermal images of iBAT temperature (left), iBAT temperature statistics (middle), and average iBAT temperature (right) of ChR2 and EGFP mice after light stimulation. **D** Changes in core (left) and average core (right) temperatures of ChR2 and EGFP mice after light stimulation. **E** Schematic diagram illustrating optogenetic inhibition (left); NpHR expression in PVH^VGlut2^ neurons (right). **F** Food intake in fasted mice before and after optogenetic inhibition of PVH^VGlut2^ neurons. **G**, **H** Similar to **C** and **D**, except for optogenetic inhibition. *n* = 6 per group. Two-way ANOVA and Šídák’s test. Data are presented as mean ± standard error of mean (s.e.m). iBAT, dorsoscapular brown adipose tissue; PVH, paraventricular nucleus of the hypothalamus.

To clarify the regulatory effects of glutamatergic neurons on feeding behaviour and body temperature, AAV-DIO-ChR2 or the control virus AAV-DIO-EGFP was injected into the PVH of VGlut2^Cre/Cre^ mice (Fig. 4A). Specific PVH^VGlut2^ neuron activation with 473 nm blue light resulted in a significant decrease in food intake compared to that of the control group during the laser on state (*p* < 0.0001) (Fig. 4B). Thermal imaging and body temperature detection further showed that optogenetic activation of PVH^VGlut2^ neurons increased both the iBAT and core temperatures of mice (*p* < 0.0001, *p* = 0.0002) (Fig. 4C and D).

After confirming that activating PVH^VGlut2^ neurons significantly increased the metabolic capacity of mice, we further investigated whether optogenetic inhibition would have the opposite effect. NpHR inhibits VGlut2 neuron activity when excited by 589 nm yellow light (Fig. 4E). Viral expression in the PVH region was confirmed by immunofluorescence. Upon laser activation, mice with optogenetically inhibited PVH^VGlut2^ neurons showed a significant increase in food intake compared to control mice (*p* < 0.0001) (Fig. 4F), whereas iBAT and core temperatures significantly decreased (*p* < 0.0001) (Fig. 4G and H).

Collectively, these findings indicate that PVH^VGlut2^ neurons maintain energy homoeostasis by reciprocally regulating feeding and iBAT thermogenesis.

### Downstream projection site labelling of PVH^VGlut2^ neurons using anterograde tracing

**Fig. 5:**
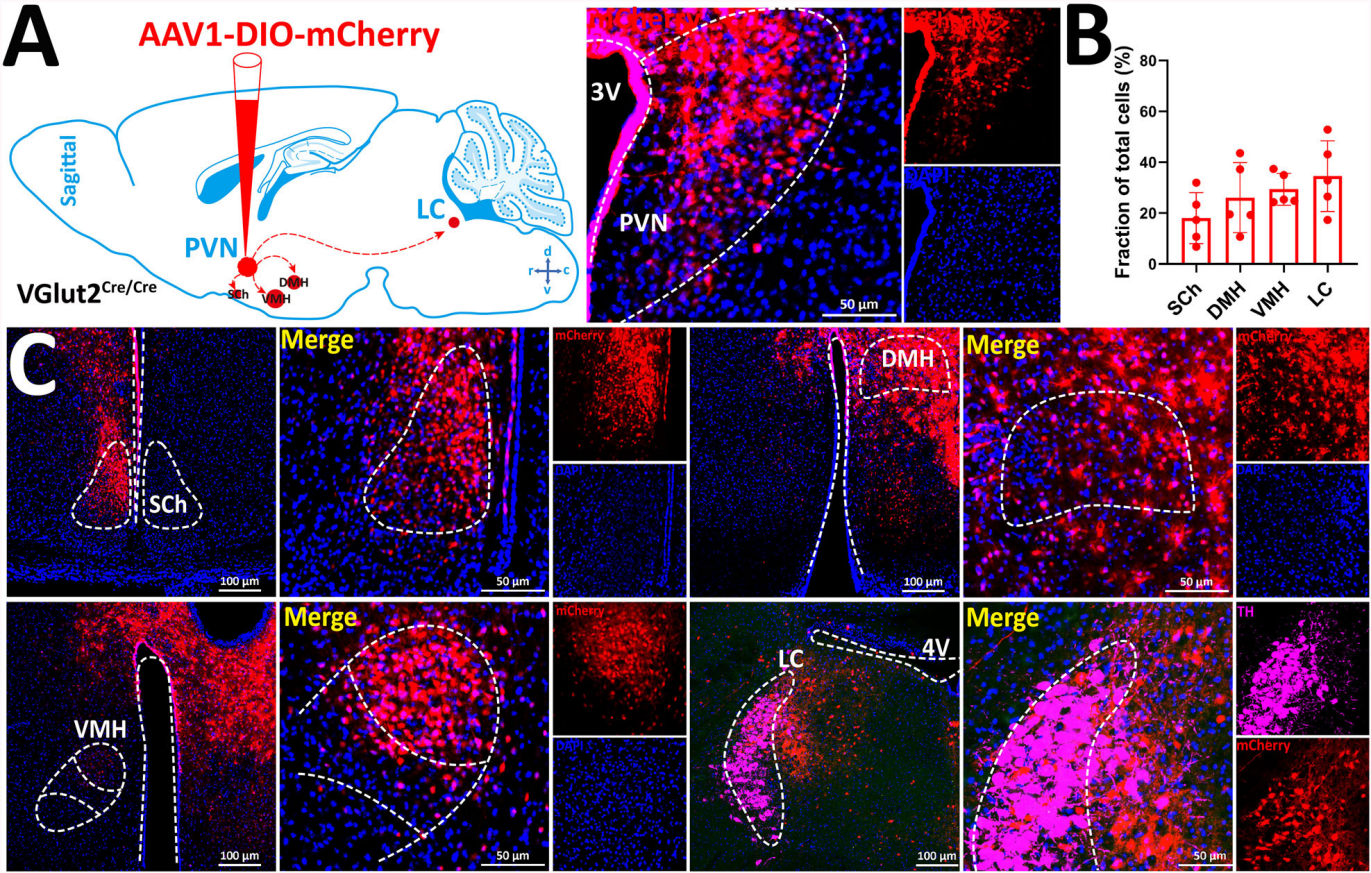
Anterograde tracing of PVH^VGlut2^ neurons. **A** Schematic diagram of virus injection (left), representative images of virus expression (right). **B** Quantitative statistics of PVH^VGlut2^ projection neurons. **C** Representative images of virus-labelled downstream output neurons. Scale bar: 100 μm. n = 5 mice. Two-way ANOVA, Šídák’s test. Data are presented as mean ± standard error of mean (s.e.m). PVH, paraventricular nucleus of the hypothalamus.

To explore the downstream targets of PVH^VGlut2^ neurons, an anterograde tracing virus, AAV1-DIO-mCherry, was injected into the PVH of VGlut2^Cre/Cre^ mice (Fig. 5A). Immunofluorescence analysis revealed that PVH^VGlut2^ neurons primarily project to the suprachiasmatic nucleus, which is involved in sleep-wake regulation. These also projected to the dorsomedial and ventromedial hypothalamus, regions implicated in fear regulation (Fig. 5B and C). Notably, numerous projection signals were detected in the LC.

Collectively, these findings suggest that the PVH → LC pathway, through its glutamatergic neurons, may significantly impact metabolic homoeostasis.

### LC^VGlut2^ neurons regulate energy metabolism in mice

To explore the regulatory effects of LC glutamatergic neurons on feeding and thermogenesis, chemogenetic methods were used to manipulate VGlut2 neuronal activity in the LC of VGlut2^Cre/Cre^ mice (Fig. S2A). Following injection of the chemogenetic agonist, clozapine N-oxide (CNO), c-Fos immunostaining confirmed successful activation and inhibition of LC^VGlut2^ neurons, thus validating effective regulation (*p* < 0.0001, *p* = 0.0007) (Figure S2B).

Compared with control mice, the food intake of mice with inhibited LC^VGlut2^ neurons significantly increased within 60 min (*p* = 0.0113, *p* = 0.0226, *p* = 0.0310). Conversely, mice with activated LC^VGlut2^ neurons exhibited significantly inhibited food intake at 15 (*p* < 0.0001), 30 (*p* < 0.0001), 60 (*p* = 0.0001), 90 (*p* = 0.0109), and 120 min (*p* = 0.0036) (Figure S2C).

Thermal imaging analysis further revealed that, in the hM3D group, CNO treatment led to increased iBAT temperature and a synchronous increase in core temperature (*p* = 0.0361, *p* < 0.0001), indicating enhanced thermogenic function. In contrast, the hM4D group exhibited decreased iBAT and core body temperatures (*p* = 0.0059, *p* < 0.0001), whereas no changes were observed in the control group (Fig. S2D and E).

Overall, LC^VGlut2^ neurons regulate both feeding behaviour and thermogenic function, thereby participating in energy homoeostasis. Their functional mode appears similar to that of PVH^VGlut2^ neurons.

### PVH^VGlut2^ → LC neural circuit regulates energy consumption

**Fig. 6:**
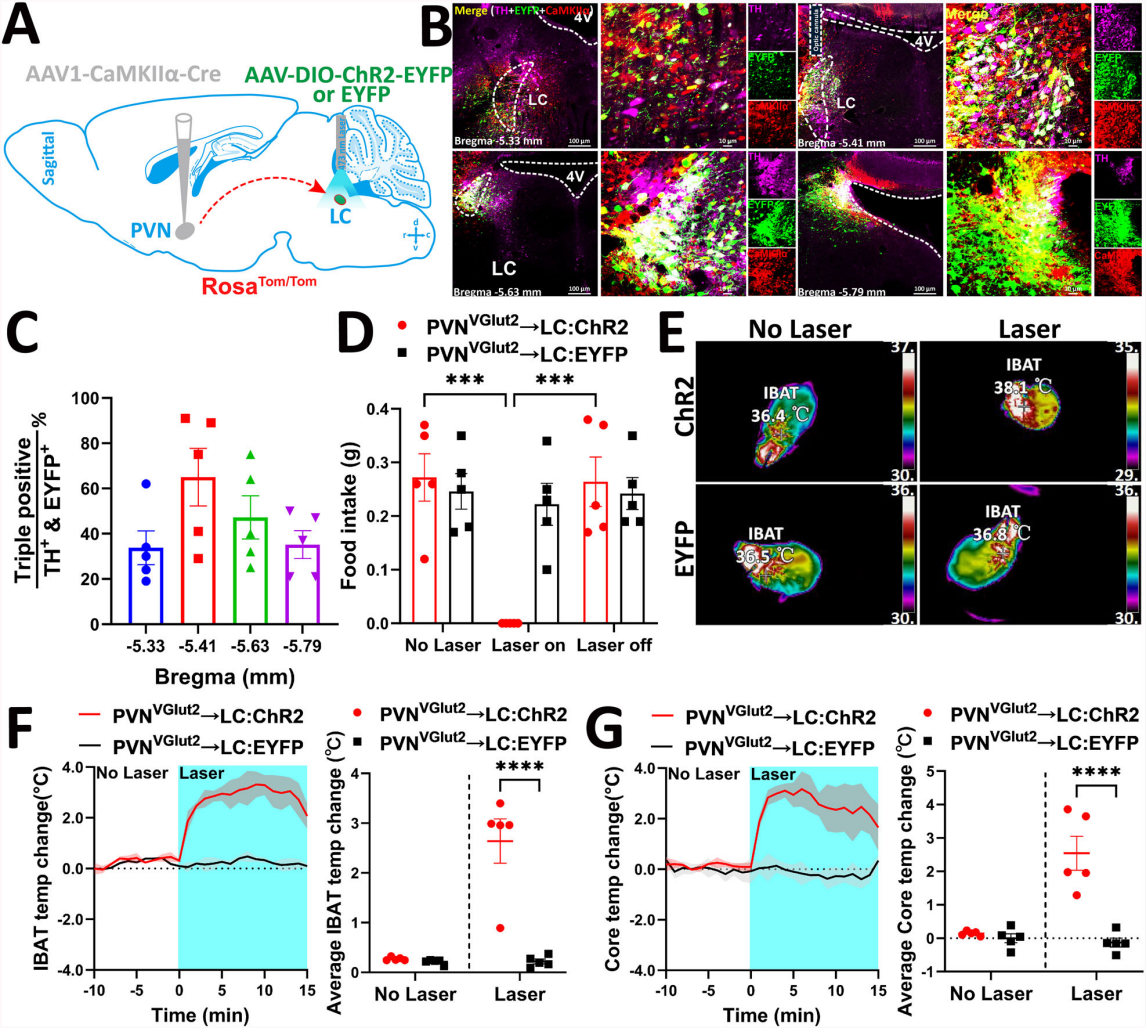
Acute activation of the PVH^VGlut2^ → LC neural circuit inhibits food intake and increases body temperature. **A** Schematic diagram illustrating virus injection strategy. **B** Coronal sections displaying EYFP expression within the LC. **C** Quantification of the proportion of EYFP-, CaMKIIa-, and TH triple-labelled positive neurons within the entire LC brain area. **D** Food intake of fasted mice before and after optogenetic activation of the PVH^VGlut2^ → LC neural circuit. **E** Representative thermal images depicting changes in iBAT temperature in ChR2 and EYFP mice before and after light stimulation. **F** iBAT temperature plots following optogenetic activation (left) and average iBAT temperature (right). **G** Similar to **F**, but showing core temperature plots (left) and average core temperature (right) during optogenetic activation. *n* = 5 mice per group. Data are presented as mean ± s.e.m. Statistical analysis was performed using two-way ANOVA with Šídák’s post-hoc test. PVH paraventricular nucleus of the hypothalamus, LC locus coeruleus, EYFP enhanced yellow fluorescent protein, CaMKIIa calcium/calmodulin-dependent protein kinase II, TH tyrosine hydroxylase, iBAT interscapular brown adipose tissue.

After confirming the metabolic regulatory capabilities of VGlut2 neurons in both the PVH and LC, we investigated whether the PVH^VGlut2^ → LC circuit itself possessed a similar function. In Rosa^Tom/Tom^ mice, an anterograde virus carrying a glutamatergic promoter and Cre enzyme (AAV1-CaMKIIα (VGlut2)-Cre) was injected into the PVH. Concurrently, an optogenetic activation virus with a DIO inverted fragment (AAV-DIO-ChR2-EYFP) and a control virus (AAV-DIO-EYFP) were injected into the LC (Fig. 6A). Three weeks post-virus expression, three-labelled neuron counting confirmed that the Cre enzyme expressed in the PVH successfully inverted the optogenetic virus and effectively targeted the entire LC (Fig. 6B and C).

Upon implanting an optical fibre in the LC and applying 473 nm blue light for activation, the food intake of mice was significantly reduced compared to that in the control group (*p* = 0.0002, *p* = 0.0003) (Fig. 6D). Optogenetic activation of the PVH^VGlut2^ → LC circuit significantly increased both the iBAT and core temperatures of mice (*p* < 0.0001) (Fig. 6E–G).

### PVH^VGlut2^ → LC circuit mediates diet-conditioned place preference

**Fig. 7:**
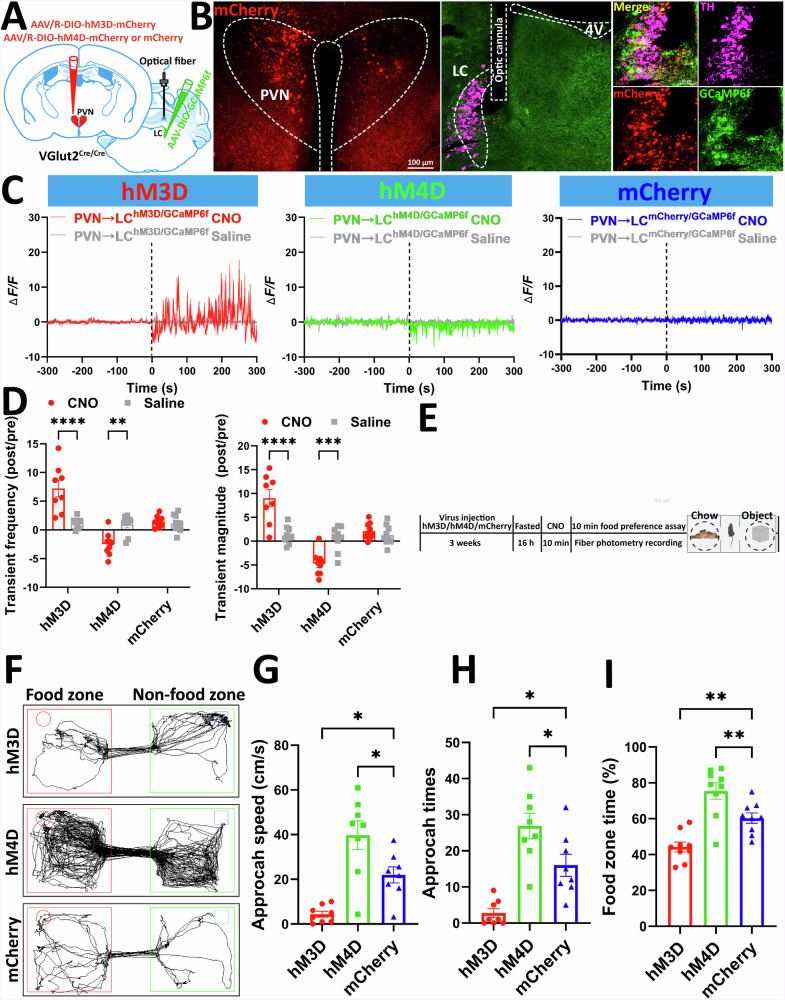
PVH^VGlut2^ → LC neural circuit bidirectionally regulates foraging behaviour. **A** Schematic diagram illustrating the virus injection strategy. **B** The left panel verifies the injection accuracy of mCherry in the PVH. The right panel verifies the expression of mCherry and GCAMP6f in the LC. Scale bar, 200 μm. **C** GCaMP6f (ΔF/F) recordings showing baseline and responses in hM3D (left), hM4D (middle) and mCherry control (right) mice before and after intraperitoneal (i.p.) injection of saline and CNO. **D** Normalised GCaMP6f transient frequency (left) and amplitude (right) in the PVH^VGlut2^ → LC circuit of hM3D, hM4D and mCherry control mice. **E** Experimental plan for the feeding behaviour preference test. **F** Track tracking illustrating foraging behaviour within 10 min when CNO activates the PVH^VGlut2^ → LC neuronal pathway. Maximum speed upon entering the food area (**G**), total number of entries into the food area (**H**), and cumulative feeding time during the 10-min feeding period (**I**). *n* = 8 mice per group. Data are presented as mean ± s.e.m. Statistical analysis: One-way ANOVA test for **D**–**F**; Two-way ANOVA with Šídák’s post-hoc test for **H**. PVH paraventricular nucleus of the hypothalamus, LC locus coeruleus, CNO clozapine N-oxide.

To clarify the direct regulatory effect of the PVH^VGlut2^ → LC neural circuit on feeding behaviour, chemogenetic manipulation combined with fibre photometry was used to modulate this circuit and monitor feeding behaviour (Fig. 7A). After immunofluorescence confirmed successful virus expression (Fig. 7B), chemogenetic virus efficacy was verified through fibre photometry.

CNO injection increased Ca²⁺ signal frequency (*p* < 0.0001) and amplitude (*p* < 0.0001) in the activated PVH^VGlut2^ → LC circuit (hM3D group), indicating enhanced neuronal activity. Conversely, CNO injection significantly reduced frequency and amplitude in the inhibited PVH^VGlut2^ → LC circuit (hM4D group) (*p* = 0.0098, *p* = 0.0037) (Fig. 7C and D), suggesting suppressed neuronal activity. No significant change in Ca²⁺ signals was observed in the mCherry virus-expressing control group, confirming chemogenetic activation and inhibition specificity.

A preference box behavioural paradigm was used to assess feeding behaviour preference (Fig. 7E). Control mice spent significantly more time in the food area than in the object area (Fig. 7F). Compared with the control group, activation of the PVH^VGlut2^ → LC neural circuit (hM3D) reduced time spent in the food area (*p* = 0.0180). Conversely, mice with PVH^VGlut2^ → LC neural circuit inhibition after CNO injection showed a significantly faster approach speed (*p* = 0.0180), more frequent food contacts (*p* = 0.0422), and significantly greater time spent in the food area (*p* = 0.0114) than control mice (Fig. 7F–I).

In summary, the PVH^VGlut2^ → LC neural circuit plays a crucial role in regulating food-seeking behaviour. Its activation inhibits the approach to and contact with food, whereas inhibition enhances food seeking.

### PVH^VGlut2^ → LC→sympathetic nervous system (SNS) circuit promotes iBAT thermogenesis

**Fig. 8:**
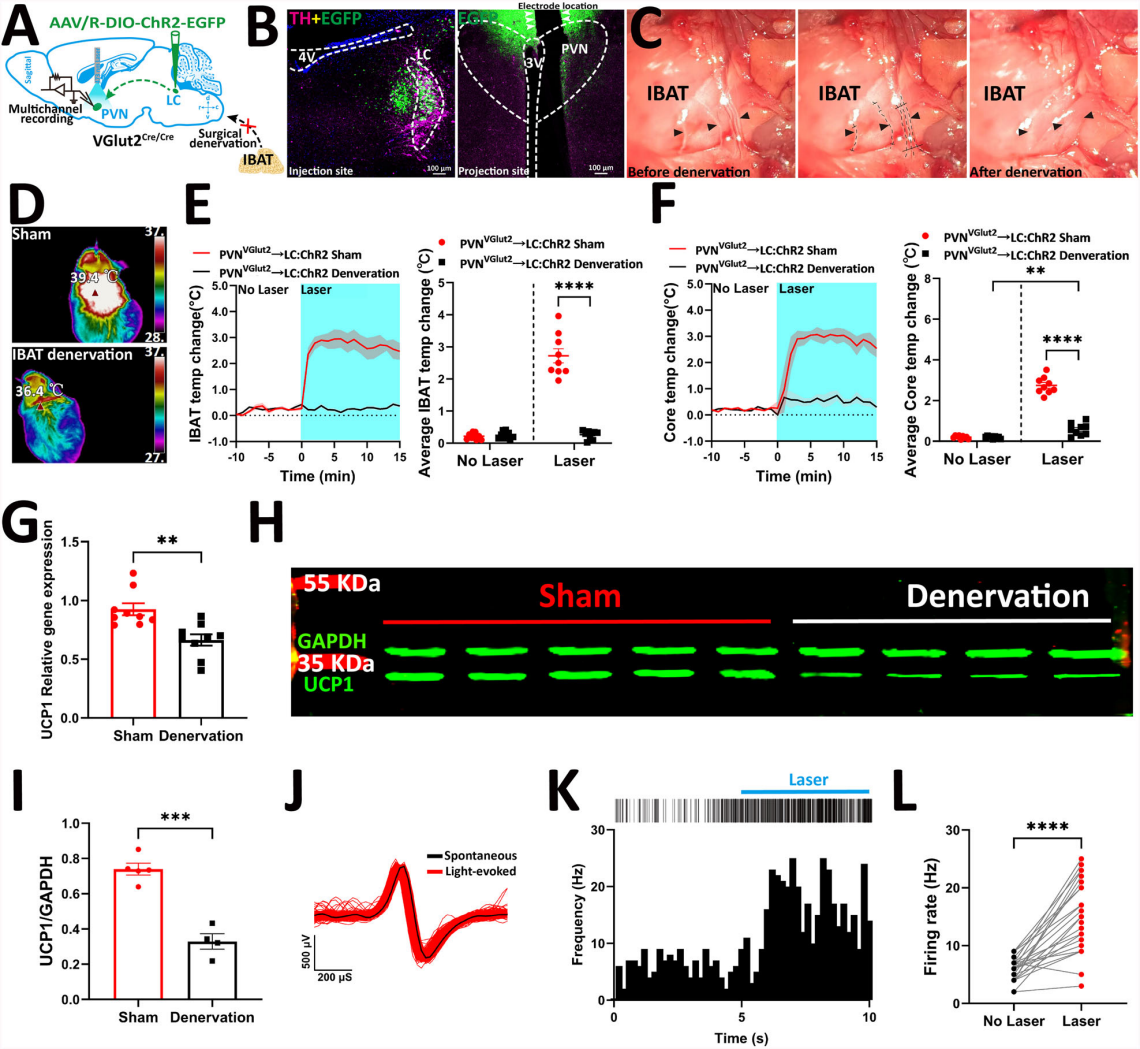
PVH^VGlut2^ → LC-iBAT circuit promotes thermogenesis and regulates body temperature through SNS activation. **A** Schematic diagram illustrating the virus injection strategy, photoelectrode implantation site, and sympathetic nerve transection experiment. **B** Verification of virus expression in the LC and electrode implantation site in the PVH. **C** Representative images demonstrating five intact sympathetic nerve branches (left); five sympathetic nerve branches after removal (middle), and the representative appearance post-denervation (right). **D** Representative thermal images depicting changes in iBAT temperature following light stimulation in sham mice (top) and iBAT-denervated mice (bottom). **E** iBAT temperature plots (left) and average iBAT temperature (right) during optogenetic activation in sham and iBAT-denervated mice. **F** Similar to **E**, but showing core temperature plots (left) and average core temperature changes (right) during optogenetic activation. **G** Relative expression level of UCP1 in iBAT after photoactivation of the PVH^VGlut2^ → LC projection in sham and denervated mice. **H** Representative western blot images showing UCP1 protein expression level in iBAT of mice in the sham and denervated groups. **I** Quantification of UCP1 protein expression in iBAT of mice in the sham and denervated groups. **J** Overlay of light-evoked (red) and averaged spontaneous (black) spike waveforms from an example unit. **K** Palisade plots (top) and PSTHs (bottom) demonstrating the increase in neuronal activity of PVH^VGlut2^ → LC neurons (*n* = 51) during optogenetic stimulation. **L** Average firing rates of a neuronal population (*n* = 26) in the presence and absence of 473 nm laser stimulation. *n* = 9 mice per group. Data are presented as mean ± s.e.m. Statistical analysis: Two-way ANOVA with Šídák’s post-hoc test for **E**, **F**; Unpaired t-test for **G**, **I**, **L**. PVH paraventricular nucleus of the hypothalamus, LC locus coeruleus, iBAT interscapular brown adipose tissue, SNS sympathetic nervous system, UCP1 uncoupling protein 1, PSTH peri-stimulus time histograms.

The SNS precisely regulates iBAT function. To clarify whether the glutamatergic circuit from the PVH to the LC activates iBAT through the SNS, functional verification was conducted using optogenetic activation combined with sympathectomy (Fig. 8A). Immunofluorescence confirmed accurate optogenetic virus injection and optrode placement (Fig. 8B). Figure 8C displays the image of mouse iBAT sympathectomy.

Thermal imaging and temperature measurements indicated that PVH^VGlut2^ → LC circuit optogenetic activation increased the iBAT temperature of sham group mice (*p* < 0.0001), whereas no change was observed in the denervated group. Thus, sympathectomy suppressed the thermogenic response to optogenetic activation. Similar to the iBAT temperature change, optogenetic activation in the sham group led to a significant increase in core temperature (*p* < 0.0001), which was relatively slower in the denervated group (*p* = 0.0037) (Fig. 8D–F).

Fluorescence quantification showed that uncoupling protein 1 (UCP1) expression in iBAT was significantly lower in the denervated than in the sham group (*p* = 0.0019) (Fig. 8G), further confirming that sympathectomy affects thermogenesis. Western blot analysis showed that UCP1 protein expression in iBAT was significantly lower in the denervated group than in the sham group (*p* = 0.0003) (Fig. 8H–I), further confirming that sympathectomy impairs thermogenic function.

In vivo multichannel electrophysiology demonstrated that optogenetic activation significantly increased light-evoked action potentials. Additionally, VGlut2 neurons exhibited a high discharge frequency and average discharge rate when the laser was turned on (*p* < 0.0001) (Fig. 8J–L), indicating effective activation of the PVH^VGlut2^ → LC neural circuit.

These findings suggest that the glutamatergic circuit from the PVH to the LC drives iBAT thermogenesis by activating sympathetic nerve output, and this effect depends on sympathetic nervous system functional integrity.

### Long-term PVH^VGlut2^ → LC circuit activation increases energy consumption and reduces body weight

**Fig. 9:**
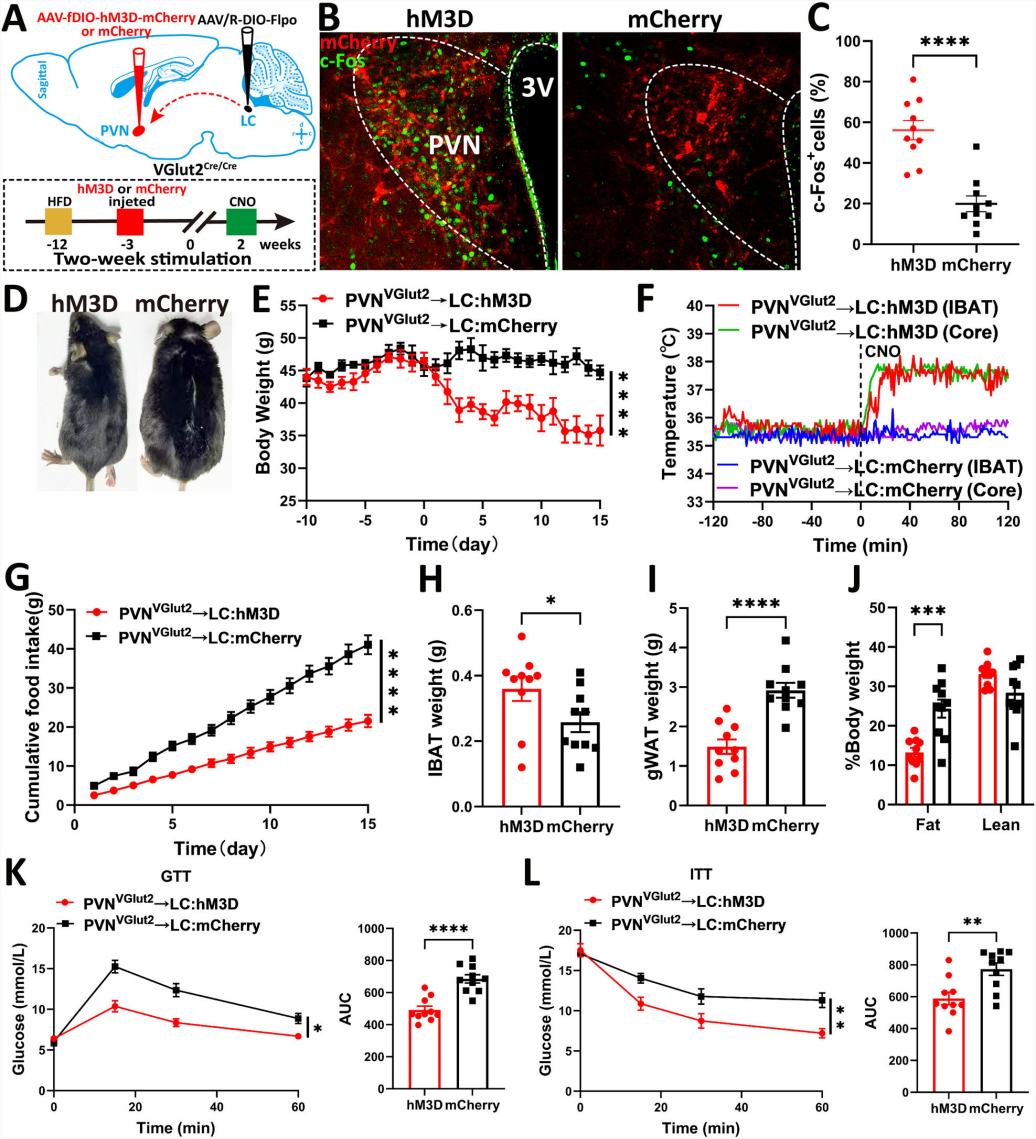
PVH^VGlut2^ → LC neural circuit improves diet-induced obesity. **A** Schematic diagram illustrating the virus injection strategy (top); Experimental scheme detailing the effect of two-week chemogenetic activation of the PVH^VGlut2^ → LC projection on the body weight of obese mice (bottom). **B** Representative images of c-Fos immunoreactivity in the LC induced by intraperitoneal (i.p.) injection of CNO in hM3D and mCherry mice. Scale bar, 100 μm. **C** Quantification of the proportion of c-Fos-positive cells among all mCherry-expressing cells. Representative images (**D**) and body weight changes (**E**) of hM3D and mCherry group mice after two weeks of chemogenetic activation. **F** Changes in iBAT and core body temperature after CNO activation of the PVH^VGlut2^ → LC neural circuit in hM3D and mCherry group mice. **G** Changes in cumulative food intake of hM3D and mCherry group mice during two weeks of chemogenetic activation. Changes in iBAT weight (**H**), gWAT weight (**I**), and body composition (**J**) of mice in hM3D and mCherry groups after two weeks of chemogenetic activation. GTT and AUC (**K**), and ITT and AUC (**L**) of mice in hM3D and mCherry groups after two weeks of chemogenetic activation. n = 10 mice per group. Data are presented as mean ± s.e.m. Statistical analysis: Unpaired t-test for **C**, **H**, **I**, **J**, **K**) AUC, (**L**) AUC; Two-way ANOVA with Šídák’s post-hoc test for **E**, **F**, **G**, **K**, **L**) PVH paraventricular nucleus of the hypothalamus, LC locus coeruleus, CNO clozapine N-oxide, iBAT interscapular brown adipose tissue, gWAT gonadal white adipose tissue, GTT glucose tolerance test, AUC area under the curve.

To explore the regulatory effect of long-term activation of the PVH^VGlut2^ → LC circuit on energy metabolism, a chemogenetic strategy was used. We activated the long-term PVH^VGlut2^ → LC neural projection in HFD-induced obese mice and assessed body weight, temperature, adipose tissue, and glucose metabolism (Fig. 9A). The retrograde virus successfully inverted the chemogenetic virus expressed in the PVH (Fig. 9B). Furthermore, c-Fos staining proved that CNO effectively activated PVH^VGlut2^ → LC neurons, verifying activation (*p* < 0.0001) (Fig. 9C).

Compared with control group mice (mCherry), mice with chemogenetic activation of the PVH^VGlut2^ → LC projection (hM3D) exhibited reduced body weight (*p* < 0.0001) and cumulative food intake (*p* < 0.0001). Additionally, the iBAT and core temperatures of hM3D mice increased, whereas the body temperature of the control group (mCherry) mice remained unchanged (*p* < 0.0001) (Fig. 9D–G).

Furthermore, chemogenetic activation increased iBAT weight (*p* = 0.0478), reduced gWAT weight (*p* < 0.0001), and decreased the fat ratio (*p* = 0.0001) (Fig. 9H–J). Consistently, mice in the activated PVH^VGlut2^ → LC projection group (hM3D) showed improved glucose tolerance (*p* = 0.0383, *p* < 0.0001) and insulin sensitivity (*p* = 0.0012, *p* = 0.0040) (Fig. 9K and L).

In summary, long-term PVH^VGlut2^ → LC projection circuit activation enhances thermogenesis and inhibits food intake, thereby ameliorating DIO and metabolic disorders.

### PVH^VGlut2^ → LC circuit glutamate release mediates energy metabolism regulation

Given the crucial role of VGlut2 neuron-released glutamate in neural signal transmission, virus tools were used to block glutamate release specifically within the PVH → LC neural circuit to explore its impact on mouse energy metabolism (Fig. S3A). Tyrosine hydroxylase staining and immunofluorescence imaging of chemogenetic virus expression confirmed accurate sensor implantation in the target area, thereby validating the experimental setup (Fig. S3B).

After CNO activated the PVH^VGlut2^ → LC circuit in the control group, an increase in glutamate release in the PVH was observed. No such response occurred in tetanus neurotoxin (TeNT) group mice (*p* < 0.0001) (Fig. S3C), confirming that glutamate release was effectively blocked. Metabolic data showed that TeNT group mice exhibited a significant increase in body weight (*p* = 0.0004), accompanied by increased food intake (*p* = 0.0006), elevated gWAT and liver weight (*p* = 0.0050, *p* = 0.0232), an increase in both the size and number of lipid droplets in gWAT (*p* = 0.0010, *p* = 0.0366), in addition to impaired glucose tolerance and insulin sensitivity (*p* = 0.0070, *p* = 0.0111, *p* = 0.0165, *p* = 0.0007) (Fig. S3D–I). Furthermore, thermogenesis in the iBAT and core body temperature decreased (*p* = 0.0100, *p* = 0.0034). WB revealed reduced UCP1 expression in the iBAT of TeNT group mice (*p* = 0.0065) (Fig. S3J and K).

In conclusion, blocking glutamate release in the PVH → LC circuit profoundly disrupted energy metabolism, leading to weight gain, fat accumulation, and metabolic disorders. These findings highlight that glutamate release within the PVH^VGlut2^ → LC neural circuit is key for metabolic homoeostasis.

## Discussion

Herein, we demonstrate that VGlut2 neurons expressed within the PVH → LC circuit bidirectionally regulate feeding and heat production. Specifically, long-term PVH^VGlut2^ → LC neural circuit activation inhibits feeding and regulates peripheral heat production, alleviating obesity.

Obesity affects central neuronal activity [18, 19]. Our findings align with previous research showing that an HFD activates ARC neurons expressing prepronociceptin (PNOC), with specific activation of ARC-PNOC neurons that promote feeding. In contrast, ARC-PNOC ablation prevents binge eating and weight gain during HFD feeding [20, 21]. Similarly, we observed increased PVH^VGlut2^ neuron activity in HFD-induced obese mice. This phenomenon may represent the compensatory negative feedback regulation in response to excess energy. However, despite this compensatory activation, obesity still develops, likely because downstream signalling is impaired by leptin resistance or synaptic dysfunction induced by chronic HFD exposure. For instance, knocking out RUVBL2-expressing neurons in the PVH (PVH^RUVBL2^) damages presynaptic vesicles, reducing excitatory transmission efficiency [22]. This suggests that an HFD could compromise compensatory effects through similar mechanisms. Optogenetic PVH^VGlut2^ neuron activation significantly inhibited food intake and reduced body weight, indicating that light stimulation may overcome HFD-induced synaptic transmission barriers. This direct enhancement of glutamatergic output from the PVH to downstream target areas could restore the inhibitory effect on food intake. This regulatory pattern suggests that PVH^VGlut2^ neurons respond to long-term metabolic stress via a negative feedback mechanism, and their activation represents a potential therapeutic target for reversing obesity [23].

Calcium signals are enhanced during foraging and rapidly inhibited during consumption, suggesting that PVH^VGlut2^ neurons promote foraging motivation but suppress this activity in response to satiety signals following food intake [24, 25]. Notably, this activity pattern is behaviourally specific, showing no change during interaction with non-food objects. This indicates that its function is specifically involved in feeding behaviour regulation rather than general motor control. Using retrograde transsynaptic tracing, we specifically identified PVH^VGlut2^ neurons projecting to the LC. Optogenetic activation of the PVH^VGlut2^ → LC projection inhibited feeding in mice, whereas inhibition of this pathway increased food intake. This aligns with reports that LC neurons regulate satiety signals [15, 17, 26], suggesting that PVH and LC achieve feeding inhibition via glutamatergic cascade pathways.

Mice with inhibited PVH^VGlut2^ → LC neuron circuits showed increased time entering and spent in the food area during place preference experiments, whereas circuit activation led to the opposite behavioural changes. This outcome may be attributed to the dense glutamatergic synaptic connections formed by PVH^VGlut2^ neuron axon terminals with LC GABAergic interneurons [27]. Enhanced PVH input to the LC can boost GABAergic activity, thereby inhibiting norepinephrine (NE) release and reducing food-seeking behaviour. Conversely, when PVH input is blocked, LC GABAergic inhibition is released, enhancing NE release and food-seeking behaviour [28, 29]. Fibre photometry and behavioural data indicate that the PVH^VGlut2^ → LC circuit may constitute a “metabolism-motivation” integration circuit. Its activation state regulates the output intensity of LC neurons, dynamically balancing energy status with feeding motivation-driven behavioural decisions.

PVH neurons regulate energy metabolism through heterogeneous mechanisms. Activating PVH^BDNF^ neurons, for example, can induce adaptive thermogenesis, increase core body temperature, and promote negative energy balance [30–32]. We found that activating the PVH^VGlut2^ → LC pathway inhibits feeding and increases iBAT thermogenesis, demonstrating that the PVH plays a core role in regulating energy metabolism behaviours. In addition to the PVH, the LC is also recognised as a key temperature regulation centre [33, 34]. These two energy metabolism-regulating nuclei form a metabolic regulation feedforward circuit via a hypothalamus-brainstem bidirectional interactive network: the PVH integrates peripheral metabolic signals to initiate food inhibition and thermogenesis activation, whereas the LC dynamically amplifies metabolic responses through sympathetic nerve output and glutamatergic signals.

Long-term chemogenetic experiments demonstrated that sustained PVH^VGlut2^-LC circuit activation significantly reduced white fat in HFD-induced obese mice, in parallel to body temperature elevation. This indicates that PVH^VGlut2^-LC circuit activation not only alters energy intake and expenditure but also influences energy storage. The glutamatergic neural connection between PVH and LC is closely related to MC4R-mediated weight regulation [35]. Long-term MC4R activation in the PVH-LC circuit enhances sympathetic nerve output [14], promoting lipolysis and energy consumption, which is consistent with the fat deposition reduction and weight reversal observed herein. Furthermore, PVH neurons play a regulatory role in glucose homoeostasis, and their excessive inhibition leads to insulin resistance and blood sugar disorders [36]. In this study, VGlut2 neuron activation within this circuit may restore positive PVH regulation on the hypothalamic–pituitary–adrenal axis, thereby improving blood sugar metabolism. Additionally, continuous circuit activation may enhance the catecholamine projection from the LC to adipose tissue, promoting white fat browning and thermogenesis, which are associated with an increased metabolic rate and adipose tissue weight changes [37]. This integrated mechanism provides a neural circuit basis for long-term metabolic improvement and underscores the potential of the PVH^VGlut2^ → LC neuronal circuit to regulate energy metabolism in mice.

Glutamate is a critical neuromodulator. Specific blocking of glutamate vesicle release within the PVH^VGlut2^ → LC neural circuit reduced the energy metabolism capacity of mice and acutely induced an obese phenotype. This phenomenon may stem from a dual imbalance of glutamatergic signalling in regulating food intake and thermogenesis. The observed increase in food intake suggests that VGlut2 neurons use glutamatergic signals to transmit inhibitory signals to the hypothalamic feeding regulatory network. When glutamate release is blocked, this appetite inhibition pathway is disinhibited, resulting in enhanced feeding behaviour. The decrease in iBAT heat production may be related to abnormal sympathetic nerve regulation mediated by glutamate. The hypothalamus regulates sympathetic nerve activity to maintain iBAT heat production, and glutamate plays a key role as the main excitatory transmitter in this pathway [38]. Following the blockade of glutamate release, rapid fat accumulation occurred, indicating that increased energy intake and reduced heat production jointly drive obesity. These findings provide crucial evidence for understanding the integrative role of the central glutamatergic system in energy homoeostasis.

In summary, this study systematically elucidates the critical role of the PVH^VGlut2^ → LC neural circuit in regulating energy homoeostasis and its underlying mechanisms. We demonstrate that this circuit coordinates feeding behaviour, thermogenesis, and lipid metabolism to maintain energy balance. These findings deepen our understanding of hypothalamic–brainstem interactions in metabolic regulation and provide both a theoretical foundation and potential therapeutic targets for developing neural circuit–based interventions against obesity and metabolic disorders.

## Supplementary information

SupplementaryFile

uncropped western blots

## Data Availability

The datasets used or analyzed during the current study are available from the corresponding author on reasonable request.
