# Supplementary material for: Paraventricular nucleus–locus coeruleus VGlut2 neural circuit regulates energy metabolism in mice
Source: Cell Death Dis. 2025 Dec 9;17(1):82. doi: 10.1038/s41419-025-08238-z (PMC12830986; doi:10.1038/s41419-025-08238-z)
Supplement: Supplementary file 1 — SupplementaryFile [file 41419_2025_8238_MOESM1_ESM.docx]

***Supplemental Material***

**Paraventricular nucleus-locus coeruleus VGlut2 neural circuit regulates energy metabolism**

**This file contains:**

Supplementary Figures 1–3, and Supplemental Method

**
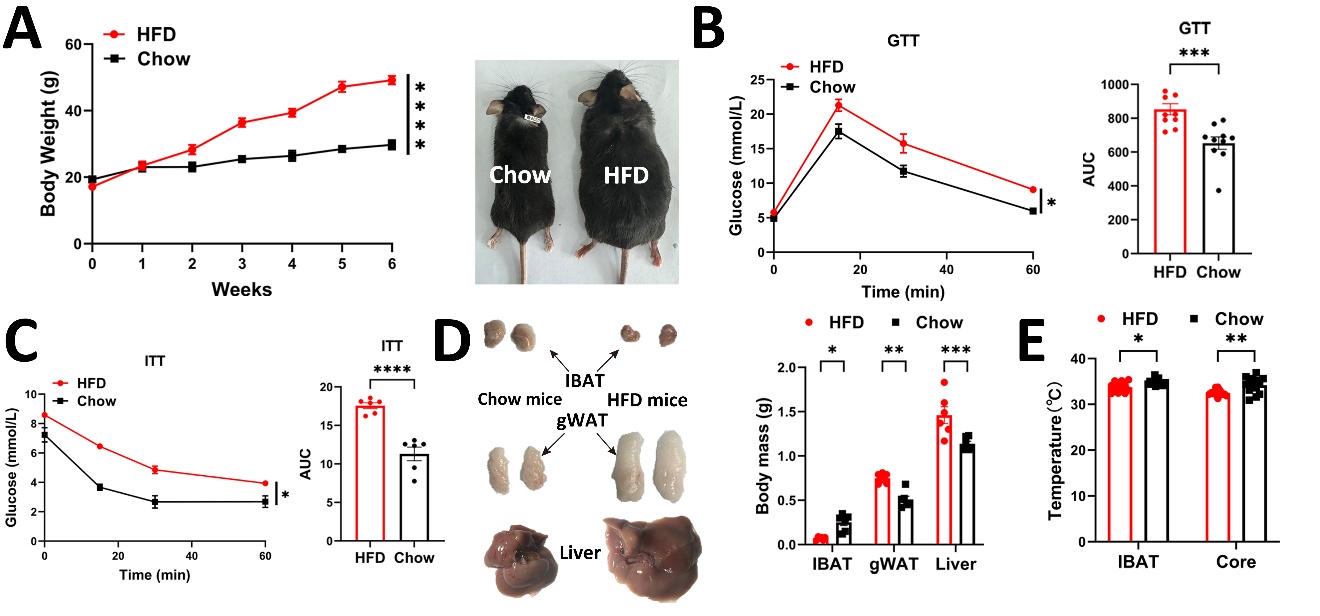
**

**Figure S1. High-fat diet (HFD) induces metabolic disorders in mice.**

**(A)** Body weight curves of mice fed with HFD or standard chow for 6 weeks (left) and representative images of mice from each group (right). **(B)** GTT results showing blood glucose levels over time (left) and AUC analysis (right) in HFD-fed and chow-fed mice. **(C)** ITT results showing blood glucose levels over time (left) and AUC analysis (right) in HFD-fed and chow-fed mice. **(D)** Representative images of iBAT, gWAT, and liver from HFD-fed and chow-fed mice (left), and corresponding tissue weights (right). **(E)** Temperature measurements of iBAT and core body temperature in HFD-fed and chow-fed mice. n=12 mice per group. Two-way ANOVA with Šídák's multiple comparisons test for (**A–C**); unpaired t-test for AUC analyses in (**B**) and (**C**); and two-way ANOVA with Šídák's post hoc test for (**D**) and **(E**). Data are presented as mean ± s.e.m. AUC, area under the curve; GTT, glucose tolerance test; HFD, high-fat diet; iBAT, interscapular brown adipose tissue; ITT, insulin tolerance test; gWAT, gonadal white adipose tissue.


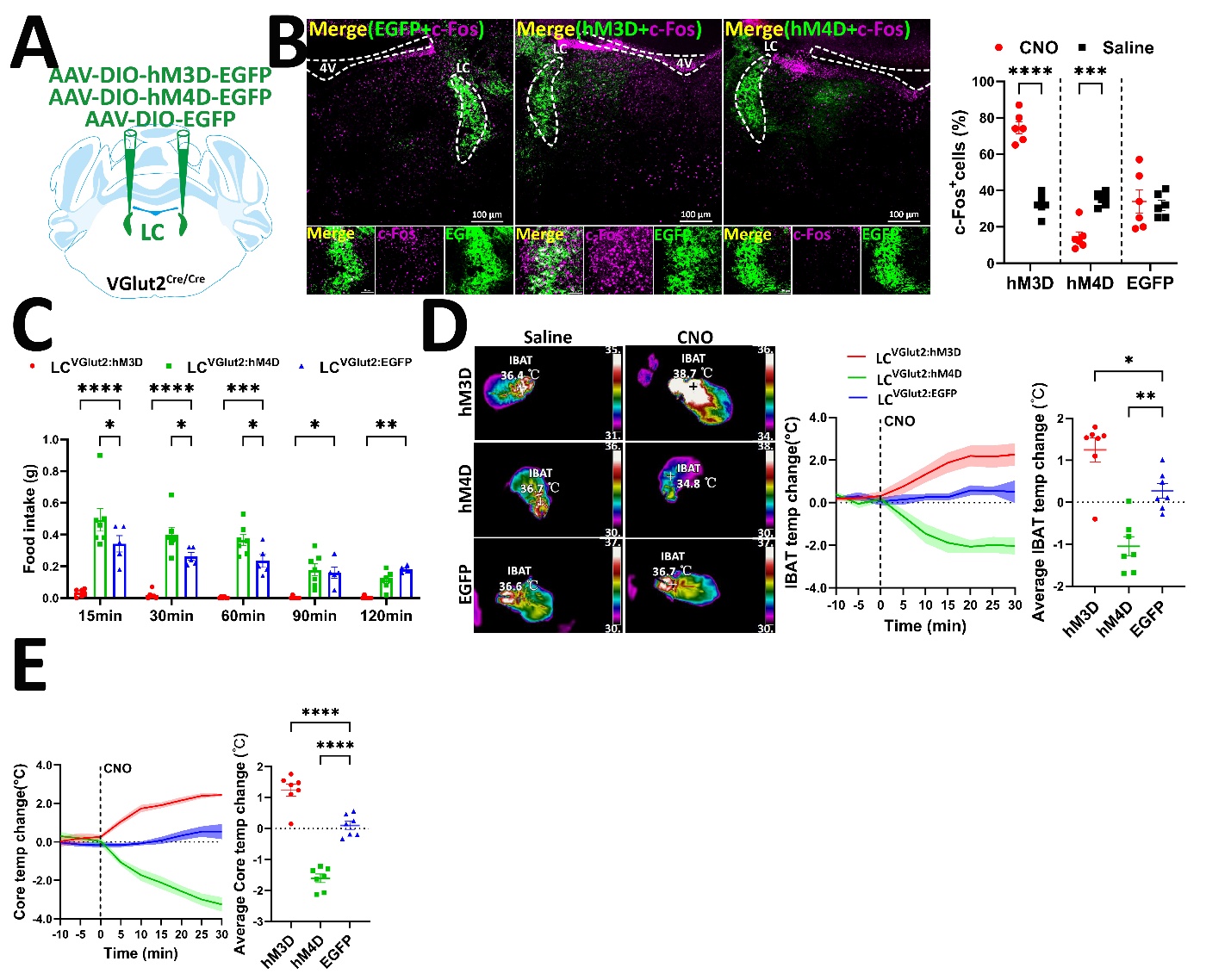


**Figure S2.** **LC^VGlut2^ neurons regulate food intake and body temperature.**

(**A**) Schematic diagram illustrating the virus injection strategy. (**B**) Representative images of c-Fos immunoreactivity in the LC after intraperitoneal (i.p.) CNO injection in EGFP-, hM3D-, and hM4D-expressing mice (left). Quantification of the proportion of c-Fos-positive cells among all cells is shown on the right. Scale bar, 100 μm. (**C**) Changes in cumulative food intake over 120 min following CNO injection in EGFP-, hM3D-, and hM4D-expressing mice. (**D**) Representative thermograms (left), individual iBAT temperature plots (middle), and mean iBAT temperature (right) of EGFP-, hM3D-, and hM4D-expressing mice 30 min after saline and CNO injection. (**E**) Similar to (**D)**, but showing representative core temperature thermograms (left) and mean core temperature changes (right) following CNO activation. EGFP n=6, hM3D n=7, hM4D n=5 mice. Data are presented as mean ± s.e.m. Statistical analysis was performed using two-way ANOVA with Šídák's post hoc test. LC, locus coeruleus; CNO, clozapine N-oxide; EGFP, enhanced green fluorescent protein; iBAT, interscapular brown adipose tissue.


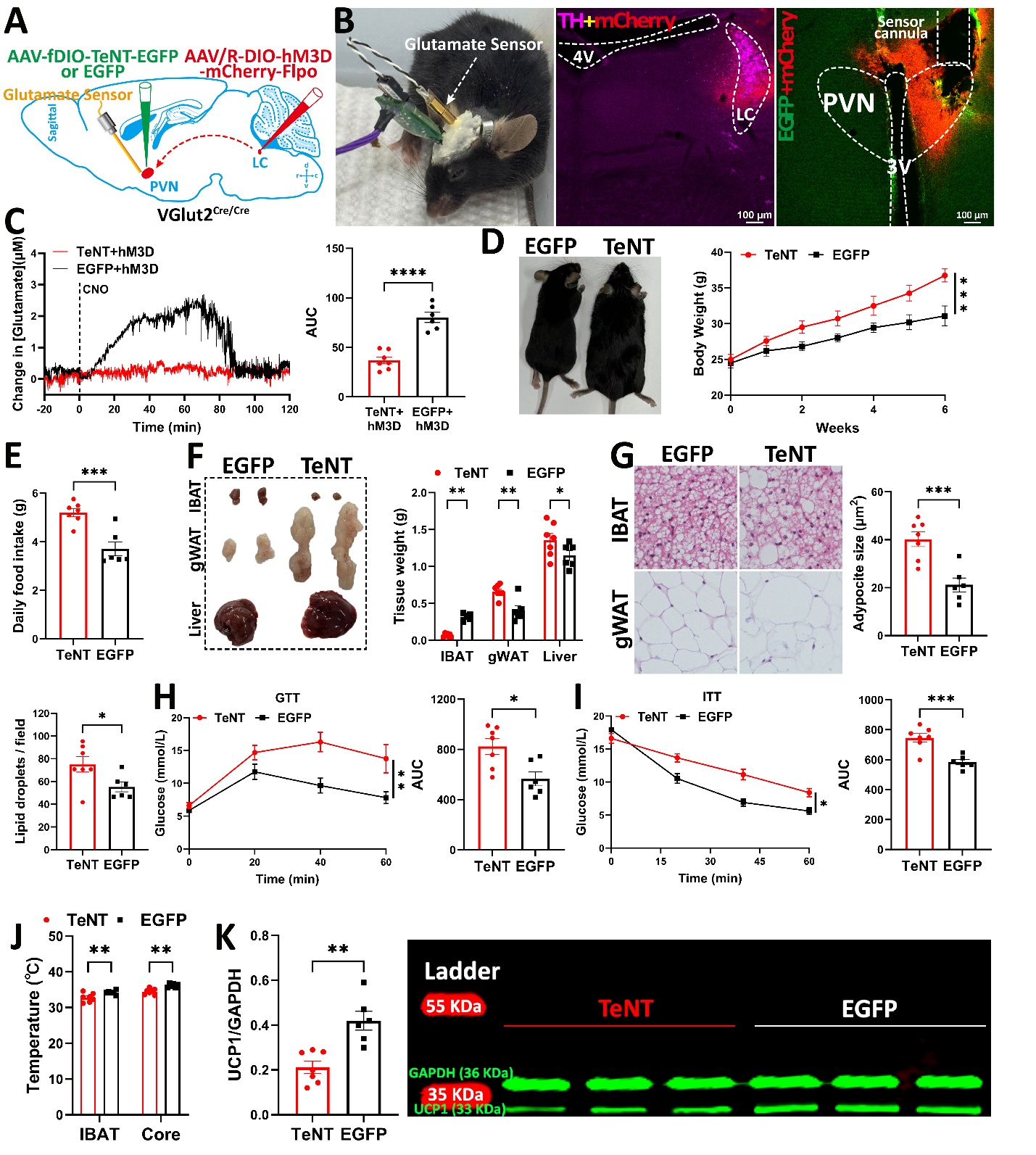


**Figure S3.** **Glutamate mediates energy balance in mice through the PVH^VGlut2^→LC neural circuit.**

(**A**) Schematic diagram illustrating the virus injection strategy and glutamate biosensor implantation. (**B**) Schematic diagram of glutamate biosensor implantation via stereotaxic injection (left); histological confirmation of LC virus expression (middle); and PVH glutamate biosensor implantation site (right). (**C**) Glutamate release (left) and AUC (right) in TeNT and EGFP group mice after CNO activation. (**D**) Representative images (left) and body-weight changes over time (right) in TeNT and EGFP group mice. (**E**) Daily food intake of mice in TeNT and EGFP groups. (**F**) Imaging of liver and adipose tissue from TeNT and EGFP group mice (left); weight of iBAT, gWAT, and liver (right). (**G**) Representative images of H&E staining of iBAT and gWAT from TeNT and EGFP group mice (left); quantification of adipocyte size in iBAT and gWAT based on histological images (right); quantification of the number of large lipid droplets in both groups (bottom). (**H–I**) GTT and AUC (**H**), and ITT and AUC (**I**) of mice in TeNT and EGFP groups. (**J**) Changes in iBAT and core body temperature of mice in TeNT and EGFP groups. (**K**) Quantification of UCP1 protein levels in iBAT of mice in TeNT and EGFP groups (left), and representative western blot images showing UCP1 protein expression level in iBAT of mice in TeNT and EGFP groups (right). TeNT group n=7, EGFP group n=6 mice. Data are presented as mean ± s.e.m. Statistical analysis: Unpaired *t*-test for AUC in (**C, F–I, K**); two-way ANOVA with Šídák's post hoc test for (**D, E, H–J**). PVH, paraventricular nucleus of the hypothalamus; LC, locus coeruleus; AUC, area under the curve; TeNT, tetanus neurotoxin; EGFP, enhanced green fluorescent protein; H&E, haematoxylin and eosin; iBAT, interscapular brown adipose tissue; gWAT, gonadal white adipose tissue; GTT, glucose tolerance test; AUC, area under the curve; ITT, insulin tolerance test; UCP1, uncoupling protein 1; CNO, clozapine N-oxide.

**SUPPLEMENTARY METHODS**

**Food intake, body weight, and tissue weight measurements**

Construction of the obesity model

Eight-week-old C57BL/6 mice were randomly divided into two groups. One group was fed an high-fat diet **(**HFD) with 60% fat (Research Diets, D12492), while the other group received a standard chow diet containing 10% fat (Harlan Teklad 2920). The mice were housed individually, and their body weight changes were monitored weekly. After 6 weeks, tissue samples were collected, and gonadal white adipose tissue (gWAT), interscapular brown adipose tissue (Ibat), and liver weights were quantitatively analysed.

Long-term chemogenetic intervention experiment

Based on the VGlut2^Cre/Cre^ obese mouse model (with a 12-week HFD modelling period), 200 nL of retrograde Cre-dependent AAV/R-DIO-Flpo virus was injected into the locus coeruleus (LC), and simultaneously, 300 nL of Flpo-dependent AAV-fDIO-hM3D-mCherry virus was injected into the paraventricular nucleus of the hypothalamus (PVH) for the experimental group, while the control group received a corresponding control virus. Three weeks post-surgery, clozapine N-oxide (CNO; 1 mg/kg, i.p.) was administered daily at 09:00 am to activate the PVH^VGlut2^→LC neural circuit. Daily food intake, body weight, and tissue weight were monitored for two consecutive weeks.

Glutamate release blockade model

A dual virus cross-regulation strategy was adopted. In VGlut2^Cre/Cre^ mice, 200 nL of retrograde AAV/R-DIO-hM3D-mCherry-Flpo virus was injected into the LC, and 300 nL of Flpo-dependent AAV-fDIO-TeNT-EGFP or the control virus AAV-fDIO-EGFP was injected into the PVH. Three weeks post-surgery, the daily food intake and body weight were recorded continuously for 6 weeks. The effect of neural regulation on fat deposition was quantitatively analysed through tissue weighing.

**Quantitative real-time polymerase chain reaction (qRT-PCR)**

Total RNA was extracted from iBAT samples using the RNeasy Mini Kit (Qiagen, Cat#74104), and its purity was determined. Subsequently, the total RNA was reverse transcribed into complementary DNA (cDNA) using the High Capacity cDNA Reverse Transcription Kit (Applied Biosystems, Cat#4368814). Quantitative real-time PCR was performed on the CFX96 system (Bio Rad CFX96).

The following primers were designed to prevent the amplification of genomic DNA: For UCP1: Forward primer: 5′-ACTGCCACACCTCCAGTCATT-3′, Reverse primer: 5′-CTTTGCCTCACTCAGGATTGG-3′. For GAPDH: Forward primer: 5′-AAGGGTGGAGCCAAAAGG-3′, Reverse primer: 5′-GGATGCAGGGATGTTCT-3′. The expression levels were calculated based on the 2^−ΔΔCt^ method.

**Western blot analysis**

The iBAT samples were initially frozen in liquid nitrogen and then ground into powder. Subsequently, the powdered samples were placed in a solution containing an ice-cold RIPA buffer (CWBIO, Cat#2333s) and protease inhibitors to prepare the homogenate. The protein concentration was determined using the BCA protein assay kit (Biosharp, Cat#BL521). A total of 20 μg of protein was separated by sodium dodecyl sulphate–polyacrylamide gel electrophoresis and then transferred onto a polyvinylidene fluoride membrane. The membrane was blocked with 5% skimmed milk and then incubated overnight at 4°C with the primary antibodies: rabbit anti-UCP1 (1:1000; ab10983, Abcam) and rabbit anti-GAPDH (1:10000; ab181602, Abcam). After washing the membrane, it was incubated with the corresponding secondary antibody, IRDye 800CW goat anti-rabbit (1:10000; 926-32211, Odyssey), at approximately 25°C for 1 h.

Following another round of washing, the membrane was detected and quantified using an infrared imaging system (Odyssey; LI-COR Biosciences). The level of UCP1 was normalised to that of GAPDH.

**Immunocytochemistry (ICC) and immunohistochemistry (IHC)**

After mice were anesthetised with isoflurane (3% for induction and 1.5% for maintenance), they were perfused with phosphate-buffered saline (PBS) and 4% paraformaldehyde (PFA) through the heart for fixation. The brains were then removed and post-fixed in 4% PFA at 4°C for 24 h. Subsequently, 50-μm coronal brain slices were prepared using a vibratome (Leica VT1200S).

The brain slices were incubated with the primary antibody (1:1000) at 4°C for 12 h. After that, they were washed three times with PBST. Then, the slices were incubated with the secondary antibody (1:2000) at room temperature in the dark for 2 h. After DAPI counterstaining for 2 min, the slices were mounted. Images were acquired using a Nikon C2 confocal microscope.

The antibodies used are as follows: Rabbit anti VGlut2 (Abcam, Cat# ab216463), Rabbit anti c-Fos (Abcam, Cat# ab209794), Mouse anti c-Fos (Abcam, Cat# ab208942), Rabbit anti TH (CST, Cat# 58844), Alexa Fluor 647 conjugated donkey anti rabbit IgG (Abcam, Cat# ab150075), Alexa Fluor 488 conjugated goat anti mouse IgG (Abcam, Cat# ab150113)

**Haematoxylin and eosin (H&E) staining**

After the iBAT and gWAT were fixed in 4% PFA for 24 h, they were washed three times with PBS. Then, the tissues underwent a gradient dehydration process using ethanol solutions with concentrations of 70%, 85%, 95%, and 100%. Subsequently, the dehydrated tissues were embedded in paraffin, and continuous sections with a thickness of 5 μm were prepared.

H&E staining was performed on the sections to visualise the morphology of adipocytes. Images of the stained sections were captured using a Nikon microscope. The size and area of lipid droplets were quantified using ImageJ software.

**Glucose and insulin tolerance tests**

Glucose tolerance test (GTT): Mice were fasted for 16 h, followed by an intraperitoneal injection of glucose at a dose of 2 g/kg. Blood glucose levels were measured using an Accu Chek Performa blood glucose meter (Roche Diagnostics) at 0 min (before injection) and 20, 40, and 60 min after injection via tail vein blood sampling.

Insulin tolerance test (ITT): Mice were fasted for 6 h, and then insulin was injected intraperitoneally at a dose of 0.5 U/kg. Blood glucose changes were monitored at the same time points and in the same way as in the GTT. The area under the blood-glucose concentration–time curve (AUC) was used to quantify all the data.

**Neural circuit tracing**

Retrograde trans synaptic tracing: In HFD-induced obese VGlut2^Cre/Cre^ mice, AAV-DIO-EGFP was injected into the PVH to visualise VGlut2 neurons. Three weeks after virus expression, the mice were anesthetised with pentobarbital sodium (50 mg/kg). After exposing the iBAT, three injection sites were selected, and pseudorabies virus PRV-RFP (2 μL per site, BrainVTA) was injected at a rate of 0.2 μL/min. The mice were then housed individually and allowed to recover for 5 days. After a 16-h fast, the mice were perfused with 4% PFA through the heart, and 50-μm brain slices were prepared. Confocal microscopy was used to collect images, and the distribution of neurons projecting from the iBAT to the PVH was analysed.

Anterograde Tracing: VGlut2^Cre/Cre^ mice were anesthetised, and AAV1-DIO-mCherry was injected into the PVH using stereotaxic techniques. After injection, the needle was left in place for a while to reduce virus backflow. The wound was sutured, and the mice were placed on a 37°C heating pad to recover and were housed separately. Three weeks after the surgery, the mice were sacrificed, and brain slices were prepared. After DAPI counterstaining, the projection areas and densities of mCherry positive neurons were observed.

**Chemogenetics**

To specifically manipulate LC^VGlut2^ neurons, 200 nL of AAV9-DIO-hM3D-EGFP, AAV9-DIO-hM4D-EGFP, or AAV9-DIO-EGFP was bilaterally injected into the LC of VGlut2^Cre/Cre^ mice (AP, −5.04 mm; ML, ±0.98 mm; DV, −3.8 mm). Three weeks after the surgery, CNO (1 mg/kg, BrainVTA, Cat#CNO-01) was intraperitoneally injected, and the changes in food intake within 2 h in fasted mice were recorded.

To chemogenetically regulate the PVH^VGlut2^→LC circuit and record the activity of PVH^VGlut2^ neurons projecting to the LC, 300 nL of Cre-dependent AAV1-DIO-hM3D-mCherry, AAV1-DIO-hM4D-mCherry, or AAV1-DIO-mCherry was injected into the PVH, and 200 nL of AAV-DIO-GCaMP6f was injected into the LC, followed by the implantation of an optical fibre to record the calcium activity of the neural circuit.

To long-term activate the PVH^VGlut2^→LC circuit, on the basis of a VGlut2^Cre/Cre^ obese mouse model (with a 12 week HFD modelling period), 200 nL of retrograde Cre-dependent AAV/R-DIO-Flpo virus was injected into the LC, and 300 nL of Flpo-dependent AAV-fDIO-hM3D-mCherry (experimental group) or control virus was injected into the PVH simultaneously. Three weeks after the surgery, CNO (1 mg/kg) was intraperitoneally injected daily at 9:00 am to activate the PVH^VGlut2^→LC neural circuit. The daily food intake, body weight, and tissue weight were monitored for two consecutive weeks.

Glutamate Release Blockade Model: A dual virus cross-regulation strategy was adopted. In VGlut2^Cre/Cre^ mice, 200 nL of retrograde AAV/R-DIO-hM3D-mCherry-Flpo virus was injected into the LC, and 300 nL of Flpo-dependent AAV-fDIO-TeNT-EGFP or control virus AAV-fDIO-EGFP was injected into the PVH. Three weeks after the surgery, the daily food intake and body weight were recorded continuously for 6 weeks. The effect of neural regulation on fat deposition was quantitatively analysed through tissue weighing.

**Optogenetics**

To analyse the function of the PVH, AAV9-DIO-ChR2-EGFP, AAV9-DIO-NpHR-mCherry, and control virus AAV-DIO-EGFP were unilaterally injected into the PVH of VGlut2^Cre/Cre^ mice. An optical fibre cannula was implanted 100 μm above the PVH to activate or inhibit VGlut2 neurons in the PVH. The mice were housed separately after the surgery for 2 weeks. Before optogenetic experiments, the mice were fasted overnight.

Activation Protocol: A 473 nm blue or 589 nm yellow laser with a pulse time of 20 ms, a frequency of 20 Hz, and a duration of 5 min was used to regulate PVH^VGlut2^ neurons. The cumulative food intake and body temperature changes were measured 5 min before, during, and 5 min after laser stimulation.

PVH→LC Pathway Activation: A dual virus system was used to specifically manipulate the PVH→LC pathway. First, 300 nL of AAV1-CaMKIIα-Cre was injected into the PVH of Rosa^Tom/Tom^ mice, and then 200 nL of Cre-dependent AAV9-DIO-ChR2-EYFP or AAV9-DIO-EYFP was injected into the LC nucleus, followed by the implantation of an optical fibre. The axon terminals were activated with 473 nm blue light, and the changes in food intake and body temperature were recorded simultaneously.

Real time Association: Cre-dependent retrograde AAV/R-DIO-ChR2-EGFP was injected into the LC nucleus, and a multi-channel optrode was implanted above the PVH. Optogenetic activation was used to associate in real time the activity of the PVH^VGlut2^→LC circuit with body temperature changes.

**Multi-channel *in vivo* electrophysiology**

The function of the neural circuit was analysed by combining optogenetic labelling with *in vivo* electrophysiology. Cre-dependent retrograde AAV/R-DIO-ChR2-EGFP was unilaterally injected into the PVH of VGlut2^Cre/Cre^ mice using stereotaxic techniques. Three weeks after virus expression, a customised 8-channel optrode array (with a fibre core diameter of 200 μm) was used. This array was surrounded by 8 platinum iridium alloy microwires, and had electrode tips protruding 200 μm from the end of the fibre. The electrode array was fixed with four titanium alloy skull screws, supplemented with cyanoacrylate adhesive and dental cement for additional reinforcement. After the surgery, the mice were kept warm and housed separately for 7 days to ensure signal stability. Subsequently, the optrode array was implanted at the original injection coordinates.

VGlut2 neurons were identified using optogenetic stimulation (473 nm, 20 Hz, 1–2 ms pulse width, 0.1–1.0 mW). Neural signals were collected at a sampling rate of 40 kHz using the Cereplex Direct system, with a band-pass filtering threshold set at 250–5000 Hz. Action potentials exceeding the baseline noise by 4 standard deviations were digitised and stored. The criteria for identifying light-responsive neurons were a firing latency of <5 ms and a spike-following rate of >90% under light-pulse train stimulation. After the experiment, the mice were perfused through the heart, and brain coronal sections were prepared to verify the electrode position.

**IBAT and core temperature analysis**

A real time thermal imager (FOTRIC 220S) was used to monitor the temperature changes on the back of the mice. To ensure the accuracy of continuous temperature measurement data in optogenetic and chemogenetic experiments, temperature probes (BioMedic Data Systems, IMI-500 Probe) were implanted in the interscapular space and abdomen of the mice simultaneously. The thermogenic response of iBAT after the activation of VGlut2 neural circuits was evaluated. Images were taken at a speed of 1 fps, with one node per min, and the average of ten images at each node was compared. For high-fat diet and glutamate release blockade experiments, only temperature probes were used to continuously monitor the iBAT and core temperatures of mice under different treatment conditions.

**Fibre photometry**

Fibre photometry was used to measure the dynamic changes of VGlut2 and VGAT neurons during the feeding process. Cre-dependent AAV-DIO-GCaMP6f virus was injected into the PVH of VGlut2^Cre/Cre^ mice, and an optical fibre cannula was implanted 100 μm above the injection site. VGAT^Cre/Cre^ mice were used as the control group and received the same operation.

After 3 weeks of recovery to ensure sufficient virus expression, the mice were placed in a free moving behavioural box with a food area (containing standard chow) and a non-food area (containing sterile wooden blocks). After the mice were allowed to adapt to the environment for 10 min, manual records of foraging and feeding behaviours (biting food for ≥2 s) and non-food interactions (nose contact for ≥1 s) and calcium signals were made. At the same time, calcium signals were collected using the fibre photometry system.

The light stimulation parameters were set as dual-wavelength alternate excitation (465 nm main signal channel/405 nm reference channel), and the output power of the optical fibre end was calibrated to 20 μW to control photobleaching effects. The original signals were analysed on the MATLAB analysis platform after baseline correction (ΔF/F = (F(t) − F_0_)/F_0_, where F_0_ is the sliding average of the signal during the 10-s period before the event). The amplitude and frequency characteristics of calcium signals within the 5 s window before and after the feeding event were mainly extracted, and the calcium transient frequency and amplitude within the 5 s before and after the feeding event were analysed. Finally, heatmaps and calcium signal trajectories were generated through cross trial averaging algorithms.

**Real-time conditioned place preference test**

To verify the connection between the PVH^VGlut2^→LC neural circuit and feeding behaviour, a behavioural test was carried out in a preference box (a white organic glass chamber with dimensions of 25×25×25 cm and an 8-cm channel in the centre). The experimental and control chambers were respectively placed with standard food and sterile wooden blocks. The mice were allowed to freely explore the chamber for 30 min to adapt to the environment before the chemogenetic operation.

Thirty minutes before the chemogenetic operation, CNO (1 mg/kg) or saline was intraperitoneally injected. The feeding-related parameters (including time spent in the food area, approach speed, and number of effective contacts, defined as nose contact ≥1 s) were quantitatively recorded using the SMART v3.0 behaviour tracking system, and the LC^VGlut2^ Ca^2+^ signals were simultaneously collected using the fibre photometry system. After the experiment, brain slices were prepared to verify virus expression and optical fibre position. Only mice with correct nucleus targeting were included in the data analysis.

**Biochemical sensor detection of glutamate release**

Biochemical sensors were used to monitor the dynamics of glutamate. A platinum-based electrochemical biosensor (0.18 mm diameter, Pinnacle Technology Inc.) was stereotaxically implanted into the PVH of VGlut2^Cre/Cre^ mice. The sensor had a platinum wire electrode as the core, and its surface was coated with a selectively permeable membrane (0.5 μm thick, which inhibited electroactive interferents) and glutamate oxidase. Glutamate was converted into α-ketoglutaric acid and hydrogen peroxide under the action of the enzyme. Hydrogen peroxide diffused through the permeable membrane and was reduced on the surface of the platinum wire, generating an amperometric current signal. The amplitude of this signal was proportional to the glutamate concentration.

A dual-channel potentiometer (Pinnacle 8400) was used to record the current signal, and the data were acquired offline after being obtained through an analogue-to-digital converter of the potentiometer. The sensitivity of the sensor was calibrated before and after each experiment using a standard glutamate solution. The original data were baseline-corrected using Sirenia Analysis (with a moving-average window of 60 s). A time window from 20 min before CNO administration to 120 min after administration was selected for signal analysis, and the noise level was controlled below ±0.5 μA. After the experiment, brain slices were prepared to verify the sensor position.

**iBAT denervation**

Mice were anesthetised with isoflurane (3% induction, 1.5% maintenance). Ibuprofen (5 mg/kg) was injected subcutaneously to relieve postoperative pain 30 min before surgery. A midline incision was made in the upper back skin, and the iBAT was exposed. The ventromedial surface of the iBAT was flipped up with microforceps to visualise the sympathetic nerves below the iBAT. Five sympathetic nerves on both sides of the iBAT (2–3 mm in length on each side) were removed with ophthalmic scissors to prevent nerve regeneration. The incision was sutured, and mice were placed on a heating pad at 37°C until fully recovered from anaesthesia, then housed individually for 7 days before experimentation.

**Statistical analysis**

All statistical analyses were performed using GraphPad Prism 9.0 software. The sample size was not predetermined using statistical methods, but it was similar to the sample sizes reported in previous publications. For each experiment, mice were randomised based on sex and body weight, and outcomes were analysed in an unblinded manner through independent assessments by more than one investigator. Samples with virus injection or optical fibre implantation deviating from the target area were excluded from the analysis. The Kolmogorov–Smirnov test was used to assess data normality and Levene's test was used to assess homogeneity of variance. For normally distributed data, parametric tests (two-sample t-test, one-way/repeated-measures ANOVA) were used, while non-parametric tests were used for non-normal data. Multiple comparisons were performed using Tukey's post hoc test after one-way ANOVA, and the Šídák correction was used for two-way ANOVA. The data are presented as mean ± standard error of the mean. A *p*-value less than 0.05 was considered statistically significant. Detailed statistical parameters for each experiment are provided in the corresponding figure legends.
