# Supplementary figures and images for: Paraventricular nucleus–locus coeruleus VGlut2 neural circuit regulates energy metabolism in mice
Source: Cell Death Dis. 2025 Dec 9;17(1):82. doi: 10.1038/s41419-025-08238-z (PMC12830986; doi:10.1038/s41419-025-08238-z)

**Figure S3K**

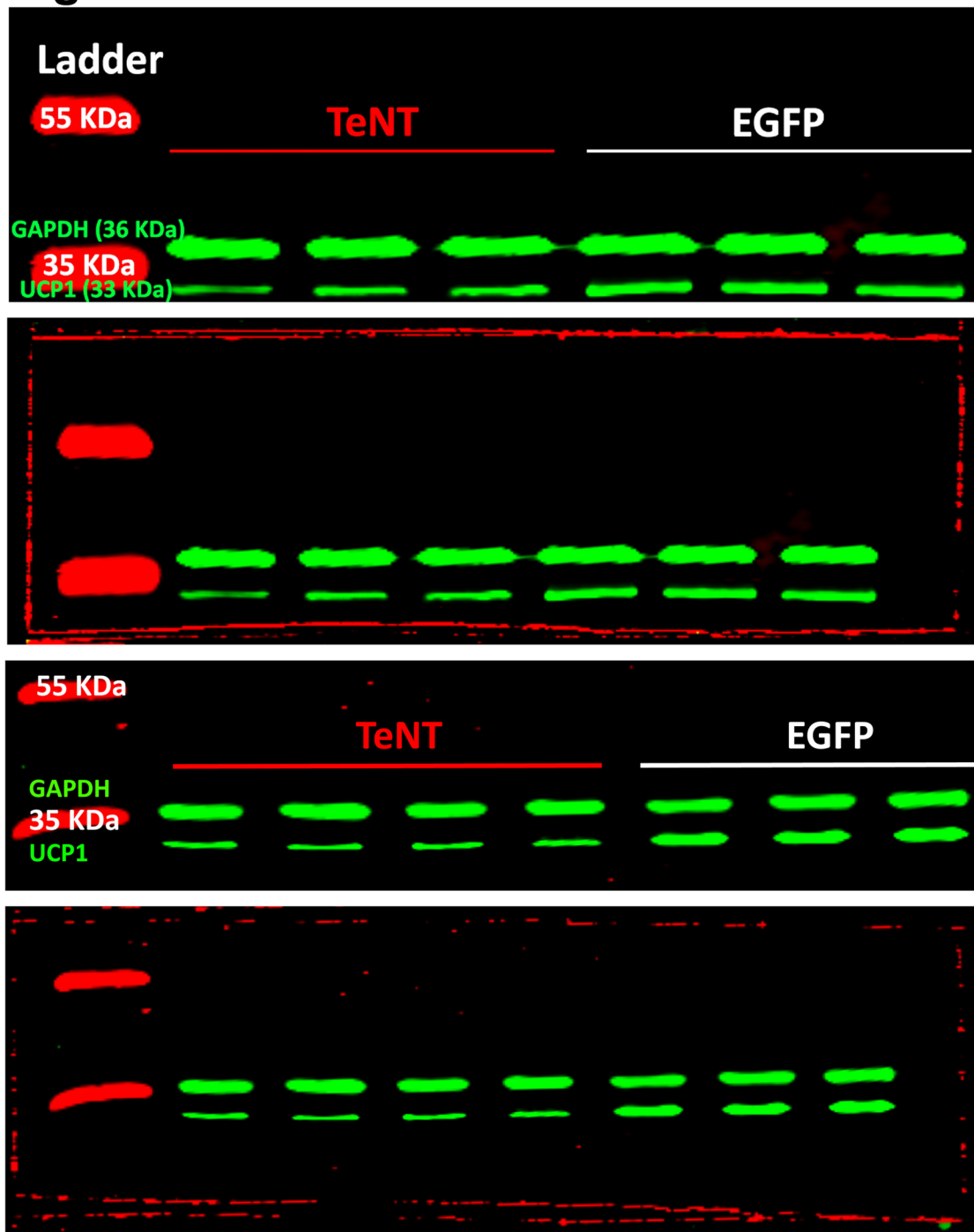

**Figure 8H**

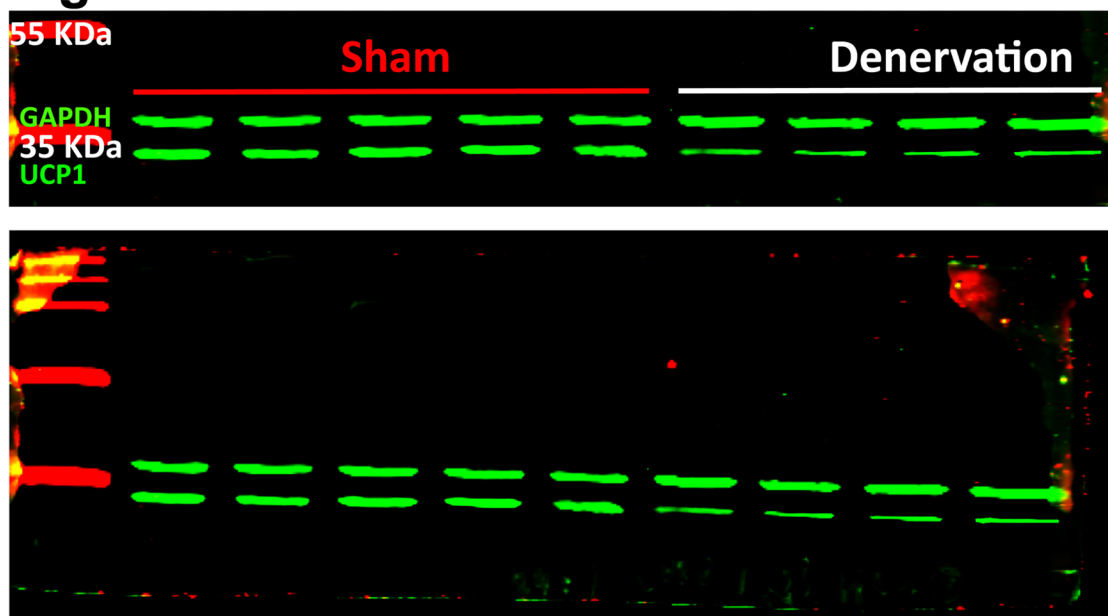

Supplement: Supplementary file 2 — uncropped western blots [file 41419_2025_8238_MOESM2_ESM.pdf]
